# Covalent Organic Frameworks: From Materials Design to Biomedical Application

**DOI:** 10.3390/nano8010015

**Published:** 2017-12-28

**Authors:** Fuli Zhao, Huiming Liu, Salva D. R. Mathe, Anjie Dong, Jianhua Zhang

**Affiliations:** 1 Department of Polymer Science and Technology and Key Laboratory of Systems Bioengineering of the Ministry of Education, School of Chemical Engineering and Technology, Tianjin University, Tianjin 300072, China; zhaofuli@tju.edu.cn (F.Z.); liu_huiming0@163.com (H.L.); mathe.denise@yahoo.com (S.D.R.M.); ajdong@tju.edu.cn (A.D.); 2 Collaborative Innovation Center of Chemical Science and Engineering (Tianjin), Tianjin 300072, China; 3 Tianjin Key Laboratory of Membrane Science and Desalination Technology, Tianjin University, Tianjin 300072, China

**Keywords:** covalent organic frameworks (COFs), nanomedicine, drug delivery, dynamic covalent bonds, biomedical application

## Abstract

Covalent organic frameworks (COFs) are newly emerged crystalline porous polymers with well-defined skeletons and nanopores mainly consisted of light-weight elements (H, B, C, N and O) linked by dynamic covalent bonds. Compared with conventional materials, COFs possess some unique and attractive features, such as large surface area, pre-designable pore geometry, excellent crystallinity, inherent adaptability and high flexibility in structural and functional design, thus exhibiting great potential for various applications. Especially, their large surface area and tunable porosity and π conjugation with unique photoelectric properties will enable COFs to serve as a promising platform for drug delivery, bioimaging, biosensing and theranostic applications. In this review, we trace the evolution of COFs in terms of linkages and highlight the important issues on synthetic method, structural design, morphological control and functionalization. And then we summarize the recent advances of COFs in the biomedical and pharmaceutical sectors and conclude with a discussion of the challenges and opportunities of COFs for biomedical purposes. Although currently still at its infancy stage, COFs as an innovative source have paved a new way to meet future challenges in human healthcare and disease theranostic.

## 1. Introduction

**Figure 1:**
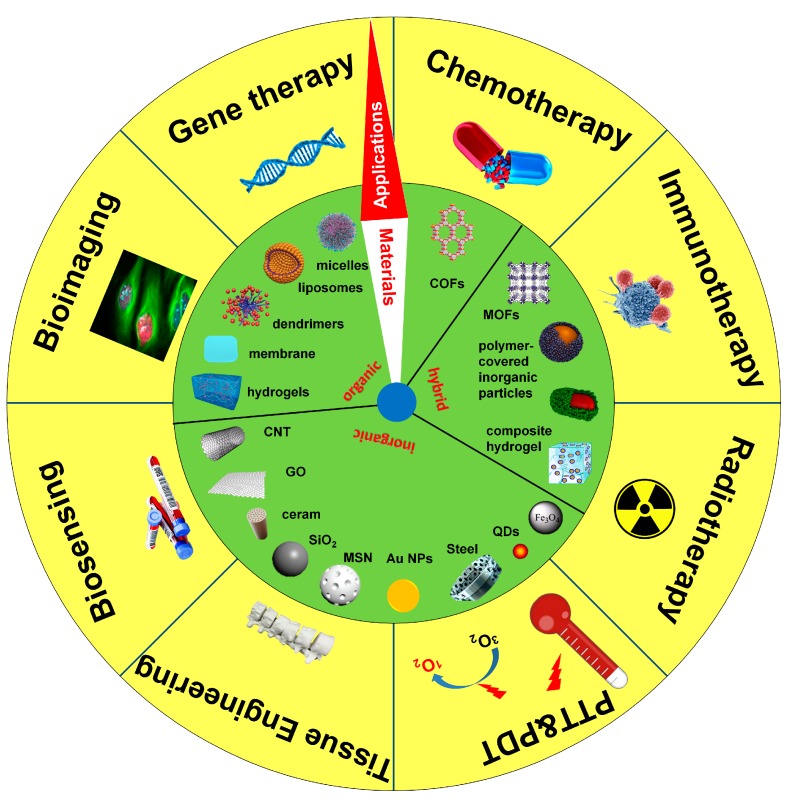
Typical materials for various biomedical applications.

Recent decades have witnessed a surge of explorations of biomedical applications in biosensing [1], bioimaging [2,3], chemotherapy [4,5], gene therapy [6,7,8,9], immunotherapy [10,11], photodynamic therapy (PDT) [12], photothermal therapy (PTT) [13,14], tissue engineering [15,16] and others [17]. Multifarious materials have been widely developed to achieve these objectives, including organic (liposomes [18,19,20,21], polymer [22,23,24], dendrimers [25,26], etc.), inorganic (metals [27], metallic oxides [28,29], carbon [30,31], mesoporous silica [32,33,34], etc.) and hybrid [35,36,37,38] materials (as shown in Figure 1). To further improve their therapeutic efficacy, researchers endow these biomaterials with stimuli responsiveness [39,40] and targeted delivery [41,42]. Some agents have been approved by the Food and Drug Administration (FDA) of the USA or in the clinical trial stage, such as Doxil^®^ [43]. However, there are still more biomaterials limited to clinical trials due to their unsatisfactory efficiency and safety [44]. Organic materials usually suffer from low in vivo stability and loading capacities [45], while inorganic substances often possess undesirable toxicity and poor degradability [46]. Metal-organic frameworks (MOFs), a kind of hybrid materials, have high surface areas, large pore sizes and good biodegradability but their chemical stability and toxicity are unsatisfactory since they are constructed by the metal coordination bond [47]. Thus, more efforts are needed to address these issues. Exploitation of new materials is one of the possible methods.

Covalent organic frameworks (COFs) are new emerged crystalline porous polymers with periodic skeletons and large surface areas [48]. Much similar to MOFs, COFs have abundant regular pores with controllable sizes and shapes and are easy to be modified but they are linked by dynamic covalent bonds as molecular building blocks, which make them not only more stable than conventional organic materials based on molecular self-assembly process but also more adaptive than inorganic particles formed by ionic or metallic bonds [49]. In addition, COFs are often composed of light atoms, such as H, B, C, N and O, often avoiding the toxicity of metal ions. This kind of composition also features COFs with low density. For example, the density of COF-18—a typical boronate ester COFs—is only 0.17 g/cm^−3^ [50].

Since first reported in 2005 [51], COFs have attracted growing attentions around the world and great developments have been scored, such as the utilization of numerous dynamic linkages to build COFs with different physicochemical properties, the improvement of synthetic methods to increase the production of COFs as well as decrease the cost, the design of pore structure to provide COFs with tunable adsorption and load capacity, the morphological control to offer different choice for various situations and the functional modification to endow COFs with more flexibilities in applications. Based on those achievements, COFs have been widely studied in heterogeneous catalysis [52,53,54], gas adsorption and storage [55,56,57], selective separation [58], semiconduction and photoconductor [59,60,61,62]. Recently, this porous framework was developed into diagnoses and treatment [63,64], since they exhibit many excellent properties including stimuli responsiveness, biodegradability, high surface area, large pore volume, tunable pore structure, π-conjugated system, unique photoelectric properties, outstanding modifiability and so on. A number of impressive reviews have summarized these developments and achievements, especially in the field of structural design and synthetic methods but so far, no review was found to specifically focus on the topic of COFs for biomedical applications. To facilitate readers to develop a better understanding of the rapid progress in the field of COFs, we highlighted the representative reviews in Table 1.

**Figure 2:**
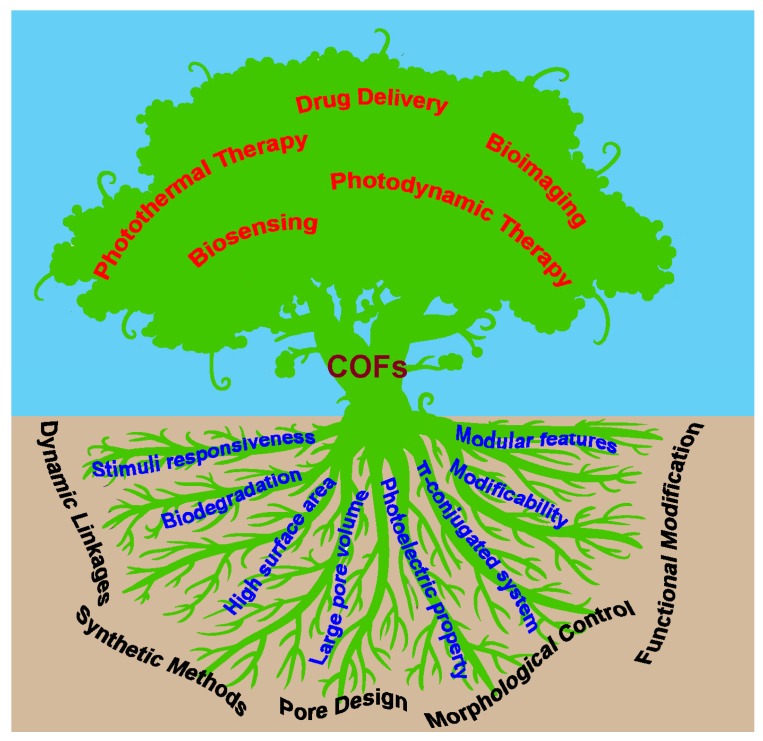
Schematic representation of the developments and properties of covalent organic frameworks (COFs) and their applications in biomedicine.

In this review, we cover the developments of COFs and summarize the applications of COFs in drug delivery, photothermal therapy, photodynamic therapy, biosensing, bioimaging and others (as shown in Figure 2). Although in the initial stage and facing many challenges, COFs have exhibited great potential to open up some new avenues for exciting opportunities in biomedical and pharmaceutical fields to improve human welfare.

## 2. Developments of COFs

The last dozen years has witnessed significant developments of COFs as can be listed: Different dynamical linkages have been used to build structures; numerous synthetic methods have been developed to prepare COFs in an easier process; pore structure can be designed as expected; various morphologies have been synthesized and several possible formation mechanisms have been proposed; multiple modification methods have been put forward and endowed COFs with more functionalities. Herein, we review these progresses and summarize the appealing features of COFs which are appropriate for biomedical applications.

### 2.1. Dynamic Likages

One of the most significant characters of COFs is their periodic two- or three-dimensional porous polymeric networks linked by dynamic covalent bonds (DCBs), which are generally formed through reversible organic reactions. Different from traditional interactions, DCBs possess robustness of covalent bonds and error-checking capability of reversible bonds at the same time [92], which endow COFs with great thermostability, multifarious adaptiveness as well as considerable crystallinity. With different dynamic linkages, COFs may exhibit diverse performance. For instance, rather robust linkages will enhance their chemical stability at the expense of crystallinity, since there is a tradeoff between the two properties [55]. In this section, we present these linkages and list their structure, stability and crystallinity in Table 2.

#### 2.1.1. B–O Linkages

The first generation of COFs are based on boron acid-based building blocks and reported by Yaghi and co-workers [51]. Boronic acid can be co-condensed with catechol to form five-membered boronate ester bond (e.g., COF-5 [51] and COF-105 [50]) and also be self-condensed to form six-membered boroxine (e.g., COF-1 [51] and COF-102 [50]). For their significant dynamic features [111], these B–O linkages exhibit outstanding error-checking capability and endow the framework with high crystallization as well as thermostability (500–600 °C). In another aspect, the reversibility of linkages makes boron-containing COFs more sensitive to water, acid even alcohols [48]. The study of Dichtel and co-workers showed that when excess H_2_O was added, HHTP-DPB COF resolved 50% within 11 s and resolved 90% within 1 min [112]. More than that, boronate ester-linked COFs can be even hydrolyzed in atmospheric moisture [65,94].

To alleviate this drawback, Yaghi and co-workers designed borosilicate-linked COFs (COF-202) by condensation of boronic acids and silanols [93]. B–O–Si bonds were rather strong and could maintain the porosity and crystallinity of COF-202 in the air for 24 h, which was a little more stable than their predecessors. Furthermore, Lavigne and co-workers took the first trial to incorporate alkyl groups into the pore walls of boronate ester-linked COFs (COF-18 Å) [113]. This modification not only changed the pore size but also protected boron from Lewis base (hydroxyl) and enhanced hydrolytic resistance of COFs. In their studies, COF-16 Å (COF-18 Å modified with ethyl groups) was chosen as sample and immerged in water for 20 min when COF-18 Å and COF-5 were used as control. The percent mass recovered result showed that 84% of COF-16 Å could be recovered while only 41% of COF-18 Å was left. BET data proved that 76% of surface area of COF-16 Å was remained while that of COF-18 Å was reduced by 95%. When dealt with same condition, COF-5, which had larger pore size than COF-18 Å, was completely hydrolyzed. Anyway, these COFs are still fragile in aqueous solution, which limits their application in aqueous medium. Zhang and co-workers reported another route to prevent boronate ester-linked hierarchical nanostructure from hydrolysis [114]. They used poloxamer 188 co-assembled with phenylboronic acid-containing monomers Im-Ba and catechol-containing monomers Im-Ca. The resulted composite nanoparticles could be stable in phosphate buffer (pH 7.4) for more than 36 h and be used to deliver doxorubicin (DOX) in vivo. Though this work did not focus on COFs, the polymer-cover method may be an effective strategy to improve the stability of boron-based COFs in water.

In 2016, spiroborate-linked ionic COFs (ICOFs) have been synthesized by incorporating cations to make boron atoms become sp^3^ hybridized boron anionic centers [94]. With this unique unit, the structure and crystallinity of these frameworks would be kept for two days in water and base solution (1 M LiOH). However, when treated with acids or stronger bases, the frameworks hydrolyzed, too.

#### 2.1.2. C–N Linkages

The most representative C–N bond linked COFs are imine-based COFs (e.g., COF-300), which were designed by Yaghi and co-workers [95]. These COFs are formed by aldimine condensation with amine and aldehyde-containing building blocks. Compared to boron-containing COFs, imine-linked COFs are more stable in water, acid and alcohols while their crystallization is lower than the former. Similarly, hydrazone-linked COFs, which are also considered as another imine-based COFs, are formed by the condensation of hydrazides and aldehydes [96]. It is believed that they possessed a better chemical stability than imine-linked COFs for additional hydrogen-bonding interactions [55]. Imide-linked COFs were prepared by imidization reaction of amines and dianhydrides and showed good hydrolytic resistance [97], which were also the first kind of COFs to be used for controlled drug release [63]. These C–N linked COFs possessed better chemical stability than B–O linked ones but are still sensitive to strong acid.

To improve the chemical stability of imine-linked COFs, Banerjee and co-workers incorporated hydroxyl (–OH) into DhaTph COFs and created intramolecular O–H···N=C hydrogen bonds [115]. The hydrogen-bond interaction preserved the structure of DhaTph COFs in water and acid (3 M HCl) for more than a week but 70% weight was lost under 3 M NaOH. They also designed another hollow spherical COF-DhaTab with similar structure, which can be stable in water, acid (3 M HCl) and Tris buffer (pH 8) for a week [116]. Jiang and co-workers proved that the incorporation of hydroxyl into 2D porphyrin COFs could enhance the interlayer interactions and trigger extended π-cloud delocalization over two-dimension sheets, which can influence the physicochemical properties of COFs [117]. Wang and co-workers reported another hydrogen bond-containing COF, Salen-COF, which could preserve its crystalline structure in the aqueous solutions at pH = 1–13 [118]. 

**Figure 3:**
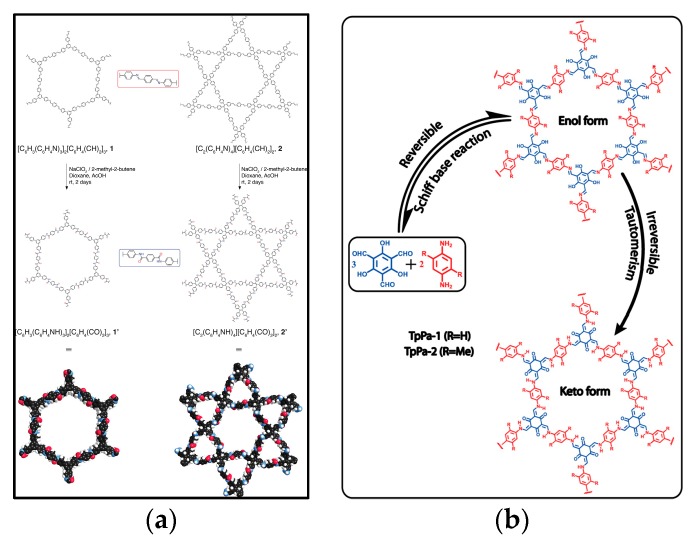
Schematic representations of conversion from imine bonds to amide bonds (**a**) and from enol-imine form to keto−enamine form (**b**). (**a**) was reprinted with permission from [98]. American Chemical Society, 2016; (**b**) was reprinted with permission from [99]. American Chemical Society, 2012.

In hexagonal imine-linked 2D COFs, each macrocycle contained 12 polarized C=N segments, which were composed of positively charged carbon and negatively charged nitrogen. Jiang and co-workers proposed that it was the electrostatic repulsion caused by these charged groups that destabilized the layered structure. Thus, they incorporated electron-donating methoxy groups into the pore walls of imine-linked COFs (named as TPB-DMTP-COFs) to reduce the electrostatic repulsion [119]. After dispersing these crystals in various solvents for a week, no obvious weight loss could be observed in under the water and organic solvents and more than 85 wt % of its original mass was remained under the condition of strong acid (12 M HCl) and strong base (14 M NaOH). Even in the boiling water, TPB-DMTP-COF still retained 72 wt %. In addition, Yaghi and co-workers converted imine bond to amide bond to improve the chemical stability of COFs after the imine-linked frameworks were prepared, as shown in Figure 3a [98]. After treated in 12 M HCl or 1 M NaOH, as-synthesized amide-linked COFs almost kept their structure through powder X-ray diffraction (PXRD) measurement.

Another method for reducing hydrolysis of imine-linked COFs is through irreversible enol-toketo tautomerism [99,120,121,122,123,124]. As shown in Figure 3b, Banerjee and co-workers used 1,3,5-triformylphloroglucinol (Tp) instead of 1,3,5-triformylbenzene to build COFs (TpPa-1 and TpPa-2) with *p*-phenylenediamine (Pa-1) and 2,5-dimethyl-*p*-phenylenediamine (Pa-2), respectively [99]. The whole process can be divided into two steps. The first step is reversible aldimine condensation, which is conducive to the formation of long-range-ordered frameworks. The second step is that the enol-imine form undergoes irreversible proton tautomerism process and becomes the keto-enamine form. With this irreversible reaction, these COFs can be stable in boiling water and acid (9 M HCl) for more than a week. What’s more, TpPa-2, with methyl groups on the pore walls, shows excellent stability in base (9 M NaOH) while TpPa-1 will lose their PXRD peaks in the first day. These new imine-based COFs (also named β-ketoenamine COFs) can also be synthesized using the mechanochemical method with a similar chemical stability [123,124]. Perepichka and co-workers prepared another β-ketoenamine COFs through Michael addition–elimination reaction of β-ketoenols and amines [125]. These crystals exhibited remarkable hydrolytic stability and could keep their structure in 9 M HCl and hot water (50 °C) for a week. Although a little decomposition was observed by PXRD when dealt with 9 M NaOH, their crystallinity is slightly inferior.

Jiang and co-workers introduced conjugation effects into COFs to improve their hydrolytic resistance. Azine-linked COFs are connected by linear and conjugated linkages (–C=N–N=C–) through condensation of aldehyde and hydrazine and show excellent stability in water, acid (1 M HCl) and base (1 M NaOH) [101]. Phenazine-linked COFs (CS-COFs) rely on their inherent π-electron conjugation effect and exhibit a similar chemical stability to azine-linked ones but their thermostability is rather robust (more than 1000 °C through TGA analysis) [102]. Squaraine-linked COFs with designed π conjugation are formed by condensation of squaric acid and copper (II) 5,10,15,20-tetrakis(4-aminophenyl) porphyrin, which are also stable in water and 1 M HCl [103]. Recently, Trabolsi and co-workers prepared viologen-based conjugated COFs based on Zincke reaction between pyridinium salts and amino groups [100]. These new kinds of COFs could be stable in boiling water and 6 M HCl for 3 days but sensitive to 1 M NaOH due to the electron transfer from the base to the viologens [104]. The conjugation effects offered these four kinds of COFs with high chemical stability but their crystallinity was moderate.

Triazine-linked COFs, also known as covalent triazine-based frameworks (CTFs), was first prepared by Thomas and co-workers through the cyclotrimerisation of nitrile building units [105]. The initial CTFs were synthesized using the ionothermal method and showed excellent thermal, chemical and mechanical stability. However, the strict condition is a great barrier for the development of CTFs and only a few cases have been reported [126,127]. Fortunately, researchers have developed new methods to prepare CTFs under mild condition [128,129,130]. Nowadays, CTFs have been developed to use as drug carriers to control release [131,132] though their crystallinity is rather low and pore size distributions are limited due to the poor reversibility of triazine linages [133].

Zhu and co-workers designed melamine-based COFs by the polycondensation of terephthalaldehyde and melamine [106]. These frameworks possessed remarkable thermal stability (400 °C) and were stable in water and common organic solvents. However, their PXRD signals were not satisfactory [134]. Recently, Yang and co-workers synthesized another melamine-based COFs based on the condensation of melamine and chlorinated triazine [135]. The surface area of the new COFs was not very large (only 301.149 m^2^/g) but their preparation was simple and cheap.

#### 2.1.3. C–C Linkages

The first C–C bonds linked COFs were prepared by Ullmann coupling reaction under ultrahigh vacuum (UHV) [136]. It can date back to 2007, when Hecht and co-workers reported a new kind of two-dimension covalently bound nanostructures formed on the surface of metals using tetra(4-bromophenyl)porphyrin molecules [136], which were considered as surface covalent organic frameworks (SCOFs) by the following researchers. After that, a number of halogenated aromatics, e.g., 1,3,5-tris(4-bromophenyl)benzene [107,137,138], *p*-bromo-benzene boronic acid [139], 4,4″-dibromo-5′-(4-chlorophenyl)-1,1′:3′,1″-terphenyl [140] and benzene-1,3,5-tricarbonyl trichloride [141], were used to synthesize SCOFs. Since C–C bonds are almost irreversible under ambient conditions, these C–C linked COFs may possess good chemical stability though no related report has been found.

C–C bonds were also applied to the construction of conjugated COFs. Jiang and co-workers prepared sp^2^ carbon-conjugated COFs (sp^2^c-COF) by C=C condensation reactions between aldehydes of tetrakis(4-formylphenyl)pyrene and acetonitriles of 1,4-phenylenediacetonitrile [108]. The sp^2^c-COF was long-term stored upon the air and stable in water, acid, base and various organic solvents.

#### 2.1.4. Other Separate and Hetero Linkages

Beside above-mentioned linkages, several other bonds were also used to construct COFs. Borazine-linked COFs are synthesized by thermal decomposition of arylamine-borane or -borontrihalide adducts, which possess high thermal stability (~420 °C) [109]. Azodioxide-linked COFs were prepared by reversible self-addition polymerizations with four tetrahedrally oriented nitroso groups [110]. This is the only monocrystalline COF that has been reported but they can lose their structures at rather low temperature (less than 130 °C). These reports focused on the chemical structures and functions, so the hydrolytic stability of these frameworks was not mentioned.

Hetero linked COFs are prepared by two or more types of covalent bonds, such as imine and boronate ester bond [142], imine and boroxine bond [143], boroxine and C–C bond [139]. It was believed that this orthogonal reaction strategy offered an alternative way to improve the functionality and stability of COFs [55].

### 2.2. Synthetic Method

No matter for which kind of materials, a facile and productive method can be intriguing and makes it possible for industrialization production. Besides that, some special processes are also needed to build ideal structures. Since Yaghi and co-workers prepared the first COF under solvothermal conditions [51], many synthetic methods have been tried in order to search for a suitable way to satisfy the needs of extensive applications. Herein, we briefly introduce the primary synthetic methods, such as solvothermal synthesis [50,95], ionothermal synthesis [105], microwave synthesis [144,145], mechanochemical synthesis [123,124], room-temperature synthesis [146,147], interface synthesis [148,149] and others [110,150].

#### 2.2.1. Solvothermal Synthesis

Solvothermal synthesis is the first and also the most widely used method to prepare COFs [66]. It is suitable to form the majority of linkages, including boronate eater [51], boroxine [51], imine [95], borosilicate [93], hydrazone [96], imide [97], azine [101], phenazine [102], squaraine [103], sp^2^-carbon [108] and borazine [109].

A classical solvothermal approach is as follows. Designated monomers are dispersed in certain solvents, which are often mixed solvents. After several freeze-pump-thaw cycles, the system is sealed to preserve the produced water molecules to keep the reversibility of the reaction. After reacted under a certain temperature (normally, from 75 to 120 °C) for several days (from 2 to 8 days), the crude products will be precipitated from solution as solid powders. The designed COFs will be obtained after washed with organic solvents, e.g., acetone and dried under vacuum. 

In solvothermal synthesis, solvents play a crucial role in the formation of COFs, which should solve the monomers but not fully dissolve them [65]. Different solvents have been used to synthesize various COFs such as dioxane/mesitylene [51], dimethylacetamide (DMAc)/*o*-dichlorobenzene [151], mesitylene/*N*-methyl-2-pyrrolidone/isoquinoline [97], ethylene glycol/aqueous acetic acid [102], *n*-butanol/*o*-dichlorobenzene [103] and so on. Jiang and co-workers explored the influence of different pairs of aromatic solvents and hydrophilic solvents on the crystallinity of boronate ester linked COFs [151]. PXRD results proved that DMAc/*o*-dichlorobenzene was the optimal combination for NiPc COF and 2:1 (DMAc to *o*-dichlorobenzene, *v*/*v*) was the best ratio. In another study, they synthesized ZnP-COF in the mixture of mesitylene and dioxane with a different ratio [152]. The crystallinity of COFs was different with the change of ratio and the best crystals were formed with the ratio of 9:1 (mesitylene to dioxane, *v*/*v*). Scanning electron microscope (SEM) images showed that the resulted particles were in uniform cubic shape under this ratio. Besides, the reaction time and the dose of catalyst also have effects on the structure of COFs, too [152,153].

It is worth noting that, the solid concentrations were generally only 2% and a great deal of organic solvents were needed in the solvothermal synthesis process, which may cause environmental pollution [154]. To solve this issue, Banerjee and co-workers used water to replace organic solvent and proved a viable, greener route to produce β-ketoenamine COFs [155]. But this method has not been testified to produce other COFs.

Since the crystalline COFs are controlled by thermodynamic equilibrium, solvothermal synthesis can provide the system with enough energy to pass the barrier of Gibb’s free energy and form crystals more easily [55]. But the synthetic conditions are strict and reaction time is long, which make it hard to use for scale-up production.

#### 2.2.2. Ionothermal Synthesis

Ionothermal synthesis was used to prepare triazine-linked COFs and was first reported by Thomas and co-workers [105]. Normally, aromatic nitrile as building blocks (e.g., 1,4-dicyanobenzene, 2,6-dicyanopyridine, 1,3,5-tris(4-cyanophenyl)benzene) are dissolved in molten ZnCl_2_ at 400 °C and reacted for 40 h. In this process, ZnCl_2_ works as solvent as well as catalyst, so that the cyclotrimerisation reaction becomes reversible. When the system is cooled down to ambient temperature, the salts are washed out and the crystalline CTFs are prepared after purification.

Compared to boronate-ester or imine linked COFs prepared by solvothermal synthesis, CTFs show poor crystallinity since the conditions of reversible reaction are very harsh. Besides that, only a few monomers are suitable for this method because the monomers should have good solubility in ionic melt and will not be decomposed under this high temperature [66]. Thus, ionothermal synthesis is not a good choice for mass production and researchers have been working hard to attempt new methods to build CTFs [128,129].

#### 2.2.3. Microwave Synthesis

Microwave synthesis has a long history in organic chemistry [156] but it was not until 2009 that this method was used in preparing COFs by Cooper and co-workers [144]. The primal process was partially similar to solvothermal synthesis. Monomers were dissolved in mixed solvent and sealed with nitrogen. And then, the system was heated by microwave irradiation and kept at 100 °C for 20 min. After purified, the COF-5 was synthesized and had comparable physical properties with that prepared by traditional way but the reaction time was shortened more than 200 times. Moreover, the BET surface area of the new-formed COF-5 (2019 m^2^/g) is a bit higher than that prepared via solvothermal method (1590 m^2^/g) [51], which is probably because microwave can effectively remove the residues or preserve the porosity. Moreover, this method was also suitable for unsealed system and could be used for both 2D and 3D COFs (COF-102) [144]. In addition of boron-based COFs [157,158], microwave synthesis has been explored to form β-ketoenamine [159,160], triazine [128] and melamine [106,134] based COFs.

Compared to solvothermal synthesis, microwave synthesis uses less time and removes impurities more effectively, showing an attractive prospect.

#### 2.2.4. Mechanochemical Synthesis

In general, building blocks are dissolved in solvents to prepare COFs, which can be limited by the physicochemical properties of monomers and the dissolving capacity of solvents. The residual organic solvents may also feature the products with toxicity [161]. Mechanochemical synthesis, a traditional process technology on industrial scale, may be an alternative choice to avoid these drawbacks [162,163]. In the last years, mechanochemical method has been used in the preparation of porous organic polymers (POP), including COFs [91].

**Figure 4:**
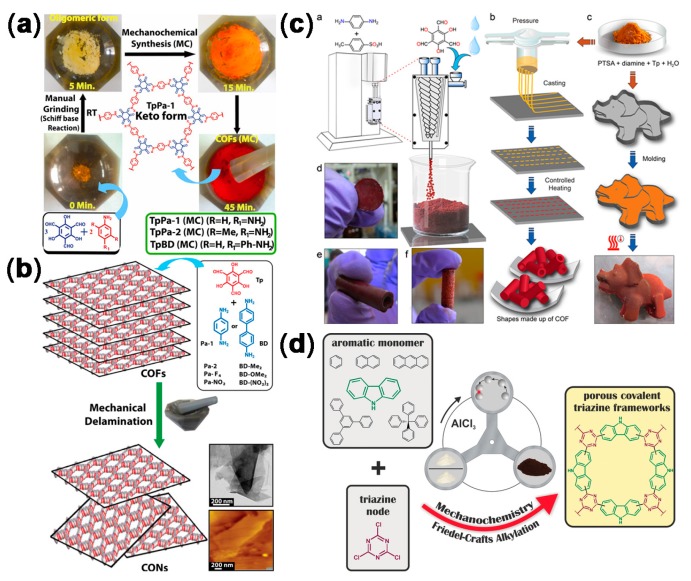
Schematic representations of mechanochemical synthesis. (**a**) Manual grinding to prepare β-ketoenamine-linked COFs, reprinted with permission from [123]. Copyright American Chemical Society, 2013; (**b**) Manual grinding to produce nanosheets, reprinted with permission from [120]. Copyright American Chemical Society, 2013; (**c**) Extrusion and terracotta technique to prepare COFs with desired shapes and sculptures, reprinted with permission from [124]. Copyright American Chemical Society, 2017; (**d**) Ball-milling to prepare CTF, reprinted with permission from [130]. Copyright John Wiley and Sons, 2017.

In 2013, Banerjee and co-workers were the first to build β-ketoenamine-linked COFs by manual grinding in a mortar and pestle [123]. It was interesting that visual color changes could be observed and indicated the extent of polycondensation (as shown in Figure 4a). After 45 min, the color of mixtures turned to red-dark, which stood for the complete COF formation (referred to as MC COFs). In comparison to the same COFs obtained from solvothermal synthesis (referred to as ST COFs), MC COFs showed similar chemical stability but their crystallinity was moderate. To alleviate such shortcomings, liquid-assisted grinding (LAG) method was applied to synthesize imine, β-ketoenamine and hydrogen-bonded imine-linked COFs [164]. Mechanical grinding also can be used to produce covalent organic nanosheets (CONs) from bulk COFs, as shown in Figure 4b [120]. The prepared CONs retained their structural integrity and be stable in water, acid and base as before exfoliated. Recently, Banerjee and co-workers developed a newly mechanochemical method to continuous produce COFs with a twin screw extruder [124]. First, diamine and 1,3,5-Triformylphloroglucinol monomers were added into *p*-toluene sulfonic acid (PTSA) in sequence and mixed well by grinding. And then, a little water was added to make the mixture converted into dough. After heated at 170 °C for 1 min, the deep reddish powders were dipped into hot water to separate the designed COFs. These COFs featured highly crystalline, ultrahigh porosity and high surface area. What’s more, COFs will be molded into any desired shapes and sculptures through heating, mimicking the ancient terracotta process, as shown in Figure 4c. This approach is widely suitable for imine-linked COFs.

As mentioned above, CTFs were synthesized by ionothermal method in the beginning [105]. Later, Cooper and co-workers developed microwave synthesis and room temperature strategy with the help of strong Brønsted acid catalyst [128]. But the operating conditions and product yield are still unsatisfactory. Banerjee and co-workers expanded mechanochemical synthesis to construct CTFs [130]. Instead of the conventional cyclotrimerisation of nitriles, the new approach is based on Friedel-Crafts alkylation. The process is described in briefly as follows. Aromatic monomers (e.g., carbazole), triazine nodes (cyanuric chloride), activating reagent (AlCl_3_) and bulking agent (ZnCl_2_) were mixed and milled in a planetary ball mill for 1 h, as shown in Figure 4d.

In brief, mechanochemical synthesis is a user-friendly, eco-friendly and time-saving alternative to produce COFs in a massive scale. It provides us a powerful method to realize the industrial production of COFs.

#### 2.2.5. Room-Temperature Synthesis

Room-temperature synthesis was first mentioned in the review of Wang and co-workers [66], though their research paper was not published until 2017 [146]. In their approach, precursor monomers were dissolved in the same solvents as solvothermal method and acetic acid was added as catalyst. Kept undisturbed at room temperature for 3 days, the imine based COFs formed and precipitated from solution. Further, Zamora and co-workers used *m*-cresol or DMSO as solvent instead of traditional mixture solvent and prepared RT-COF-1 in minines [165,166]. Later, they combined this methodology with microfluid technology and constructed COFs in fibrillar micro-structures [167]. When Sc(OTf)_3_ was used to replace acetic acid, the frameworks were synthesized within 10 min and possessed a large specific surface area [147].

Room-temperature synthesis avoids strict reaction conditions but only –C=N– linked COFs have been prepared by this way. The generalization of this facile route still remains challenges.

#### 2.2.6. Interface Synthesis

Interface synthesis is a bottom-up method to prepare COF films at the interface of solid-liquid, solid-vapor, liquid-liquid and liquid-air interface.

**Figure 5:**
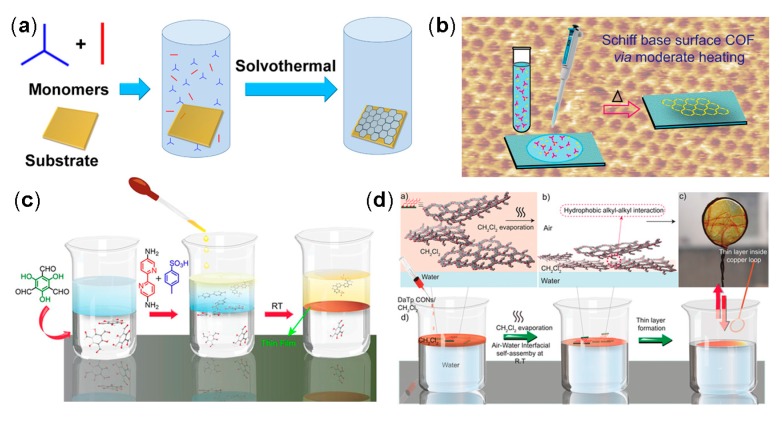
Schematic representations of COFs synthesis at the interface between (**a**) solid-liquid; (**b**) solid-vapor, reproduced with permission from [177]. Copyright American Chemical Society, 2013; (**c**) liquid-liquid, reproduced with permission from [178]. Copyright American Chemical Society, 2017; (**d**) liquid-air, reproduced with permission from [179]. Copyright John Wiley and Sons, 2016.

Solid-liquid interface synthesis is often assisted with solvothermal method (as shown in Figure 5a) [148]. Normally, precursors are suspended in a suitable component solvent. Then, substrate is added into the mixture solution, sealed and heated for a certain time. After washed and dried, the 2D layered COFs will be collected on the surface of substrate. The substrate can be Au [168], highly ordered pyrolytic graphite (HOPG) [169], single-layer graphene (SLG) [148,149,170,171,172], three-dimensional graphene (3DG) [173], glass [174] and silicon [174,175]. In particular, Dichtel and co-workers extended this method to flowed solution [176]. By this way, COFs films will be synthesized with reduced roughness though large amounts of monomers are abandoned. Further, this strategy can also be executed under room-temperature condition [165].

Solid-vapor interface synthesis is often used by combination with ultrahigh vacuum (UHV) method [107,136,137,138,139,140,180,181]. Halogenate monomers will be turned into vapor at first and deposited on the surface of clean metal surface under ultrahigh vacuum. Ullmann coupling reaction and annealing process will be conducted under high temperature. Besides, this method is also suitable for polyester condensation [141]. Another way to build COF layers at the interface of solid-vapor is through drop-casting [177,182,183,184,185]. As shown in Figure 5b, a little of precursor solution is dropped on the surface of substrate and the solvent is removed through evaporation. The third route is named as vapor-deposited method [186]. One of precursors was preloaded onto the substrate at first and the other was induced with vapor. With CuSO_4_·5H_2_O regulating the water content, atomic thick sheet would be obtained on the solid surface. Beside imine-linked COFs, this approach was suitable for boroxine-linked and imine-boroxine hybrid linked COFs, too [187,188].

Liquid-liquid and liquid-air interface synthesis are both used to prepare free-standing films that can be transferred onto other substrates [178,189,190,191,192]. Liquid-liquid interface method is shown in Figure 5c [178], monomers and catalysts are dissolved in two immiscible solvents and the stratified laminar between two immiscible solvents, i.e. interface layer, will be formed. Monomers are polymerized and transformed into thin film at the interface. Xu and co-workers prepared triazine-based COFs at the interface of dichloromethane and trifluoromethanesulfonic acid [191]. Assisted with reflux, the single-layer or few-layer free-standing films would be synthesized with large area and high crystallinity. Classical liquid-air strategy is known as Langmuir-Blodgett method, as shown in Figure 5d. Very little of monomers solutions are added on water in order and the organic solvent is removed carefully. The result mixtures are kept for several hours for polymerization. This approach has been widely used in synthesis of free-standing ultrathin covalent organic nanosheets even monolayers at the air/water interface [179,192].

Apart from films, interface synthesis is also used to construct frameworks on the surface of other particles, such as Fe_3_O_4_ [193,194] and MOF [195,196].

Interface synthesis is an ideal strategy to prepare well-structured COFs and great progress has been made in the past several years. However, the production capacity of this method is rather low.

#### 2.2.7. Other Synthetic Methods

Beside above-mentioned methods, there are still some other ingenious approaches to build COFs.

Shortly after COFs were first synthesized under solvothermal condition [51], Lavigne and co-workers reported a reflux method to prepare COF-18 Å [197]. Briefly, the monomers were dissolved in the mixture of THF and methanol (49:1) and refluxed under nitrogen for 3 days. For without sealed vessel and under ambient pressure, the conditions of reflux synthesis are more facile than that of solvothermal synthesis. Choi and co-workers used UV-induced polycondensation of boronic acids and catechol groups to construct COF-5 without heating [150]. Results showed that reaction yield reached around 75% after 1 h and was saturated within a few hours using a xenon (Xe) lamp. The photochemical synthesis offers a new way to quickly produce COFs.

**Figure 6:**
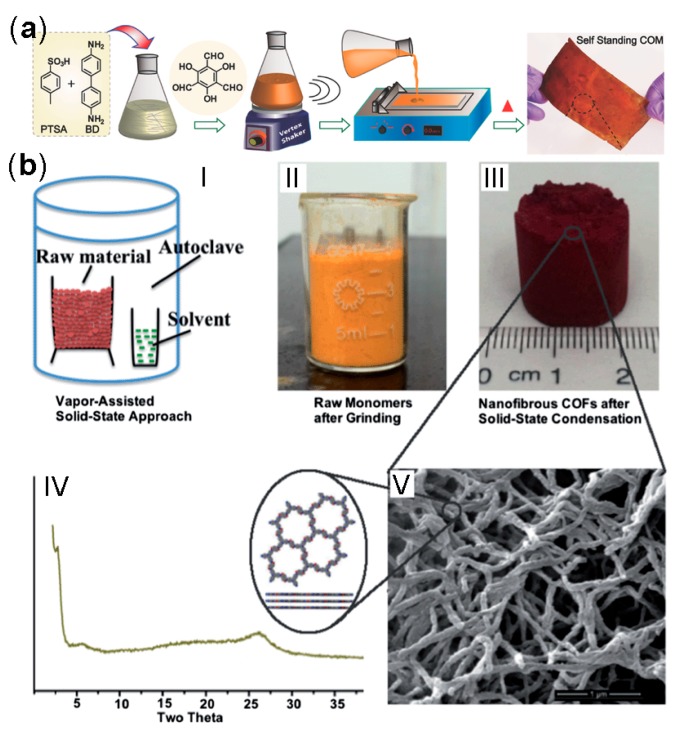
Schematic representations of (**a**) the fabrication of free-standing COF membranes, reproduced with permission from [198]. Copyright John Wiley and Sons, 2016; (**b**) vapor-assisted solid-state approach to synthesize nanofibrous COFs, reproduced with permission from [154]. Copyright Royal Society of Chemistry, 2014.

Based on their former works [99,124,164], Banerjee and co-workers developed a method to fabricate highly porous, free-standing and crystalline COF membranes (COMs) [198]. As shown in Figure 6a, monomers and PTSA were mixed with a little water and shaken until a dough was formed. Then, the dough was paved on a glass plate, covered and put into a programmed oven. The temperature was kept at 60 °C for 24 h, 90 °C for the second 24 h and a higher degree centigrade (105~120 °C, depending on the monomers) for the last 24 h. After heated, the resulting membranes were washed to remove impurities. These COMs with a large-scale length exhibited ultrahigh chemical and structural stability and may be used in water treatment and drug recovery. However, if the temperature increases faster, blister and cracks will be formed in the membranes.

Based on mechanochemical synthesis, Liu and co-workers synthesized nanofibrous COFs through a vapor-assisted solid-state approach [154]. Briefly, monomers were grinded for 1 h and exposed to solvent vapor at 120 °C for 48 h, as shown in Figure 6b. This approach is easy to handle and can also be used to form nanohybrid structures with graphene oxide.

Normally, COFs are microcrystalline powders but Wuest and co-workers prepared a monocrystalline covalent organic network by reversible self-addition polymerization [110]. The process was started from tetrakis[4-(hydroxylamino)phenyl]methane and Celite-supported silver carbonate, which was used to translate hydroxylamino to nitroso groups. After purified, the resultant precursors, which were dissolved in the mixture solution of mesitylene and ethanol (3:2, *v*/*v*) or analogous solvents, were sealed and left undisturbed under room temperature. After 48 h, the yellow crystals were collected by filtration. This approach produced large single crystals with significant porosity under mild conditions.

CTFs also can be synthesized via lower temperature polycondensation approach [129]. Based on the condensation reaction of aldehydes and amidines, Cooper and co-workers used DMSO as the solvent and cesium carbonate as a catalyst to construct CTF-HUST at 120 °C without a closed reactor or an inert atmosphere. This improvement of technology makes it possible to produce CTFs in large scale.

Besides changing reaction conditions, yield and crystallinity also can be improved by changing precursors [199,200,201,202,203]. Usually, the building blocks of imine-based and boronate based COFs, cannot be completely dissolved in organic solvents. What’s more, catechols and aldehydes are easily oxidized. These drawbacks have negative effects on the crystallinity and productivity. To address these issues, Dichtel and co-workers used protected catechols as precursors [199,200]. Using BF_3_·OEt_2_ as a catalyst, deprotection and esterification were performed at the same time to obtain COFs. Bein and co-workers used boronate ester as starting materials to build BTD-COFs through the transesterification process [201]. BTD-COFs showed pretty well physical property and the pores of these frameworks can be observed clearly by TEM. For the same reason, Yaghi and co-workers used *tert*-butyloxycarbonyl (Boc) groups to protect amine building blocks to improve the crystallinity of COFs [202]. Wang and co-workers applied dimethyl acetals and amines prepared imine-linked LZU-20, hydrazone-linked LZU-21 and azine-linked LZU-22 with highly crystalline and good thermal stability [203]. 

In conclusion, enormous progress has been done in the synthesis of COFs during the past dozen years and researchers have never ceased efforts to explore more effective ways.

### 2.3. Pore Design

COFs are characterized by high surface area and porous structure which make them ideal carriers for adsorbates [56,58], catalysts [52,53,54] and drugs [63,64]. Like other porous materials, e.g., mesoporous silica nanoparticles [34], porous polymer microspheres [204,205] and MOFs [35], the size, shape and volume of pores are important factors for cargo carry and release control. Thus, how to careful design the pore structure and keep the integrity of porosity during preparation process is very important for their practical applications. Many researchers have done lots of related studies [48,65,66,78] and we summarized the representative ones in this section.

**Scheme 1:**
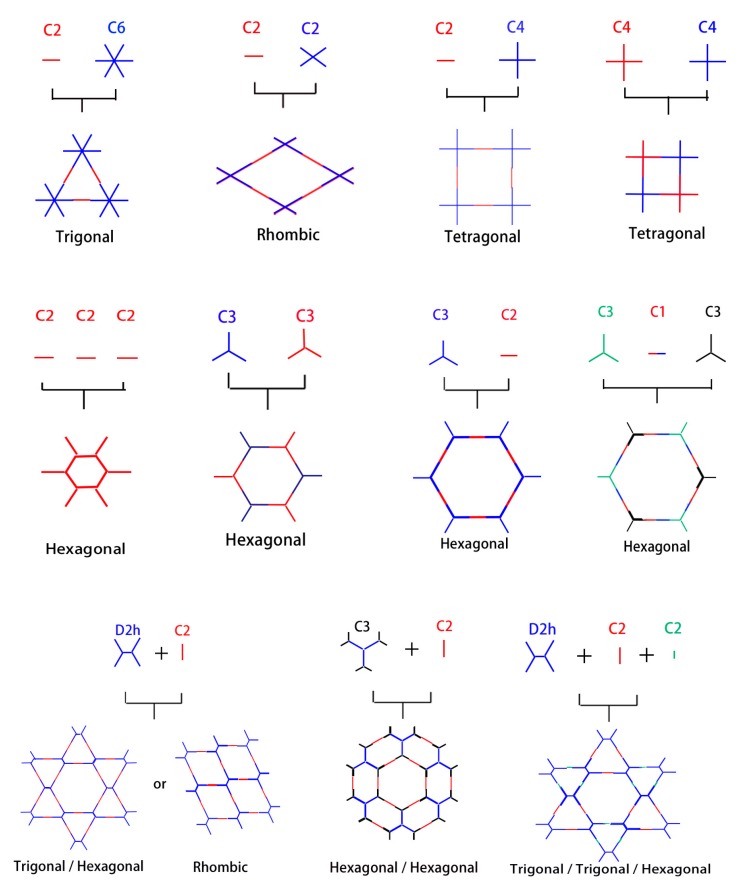
Topology diagrams of COFs representing the relationship between the pore structures and blocks.

**Figure 7:**
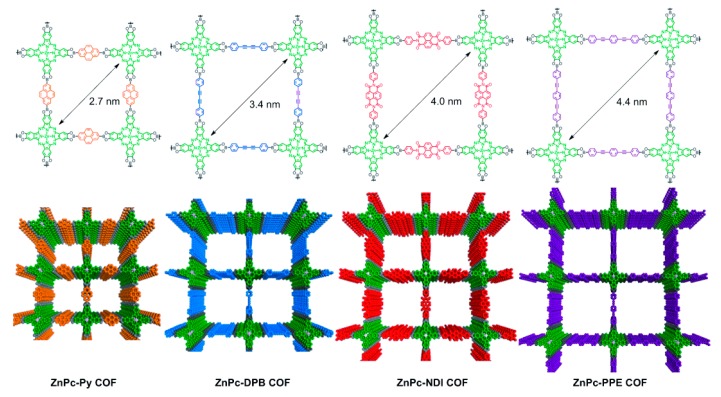
Relationships between the pore size of ZnPc COFs and different linkers, reproduced with permission from [149]. Copyright John Wiley and Sons, 2012.

For designed COFs, the shapes and sizes of pores are determined by the geometrical configuration of building blocks [66]. Jiang and co-workers proposed topology diagrams to explain the relationship between the pore structures and blocks, as shown in Scheme 1 [48,206]. As so far, pores with hexagonal [51,105,207], tetragonal [151,152], rhombic [115,208] and trigonal [209] structures have been reported. The smallest pore is only 8.6 Å and synthesized by co-condensation of 2,3,6,7,10,11-hexahydroxytriphenylene (HHTP) and 1,3,5-benzenetriboronic acid (BTBA), named COF-6 [207]. And the largest pore is 5.8 nm and belongs to PC-COF reported in 2016, to the best of our knowledge [210]. The pore size can be changed with different linkers and knots [142,149,206,207,211]. For example, when Zn octahydroxyphthalocyanine was used as knots to connect with different linear diboronic acid linkers, the pore size was changed from 2.7 to 4.4 nm (Figure 7) [149]. There are also many other large-pore COFs that have been reported, such as BTP-COF (4 nm, 2011) [145], HHTP-DPB COF (4.7 nm, 2011) [170], Star-COF-3 (4.7 nm, 2013) and D_TP_-A_NDI_-COF/D_TP_-A_PyrDI_-COF (5.3 nm, 2013) [212].

Lavigne and co-workers adjusted the pore size by incorporating alkyl chains on the walls [213]. They introduced methyl, ethyl and propyl groups into COF-18 Å and the pore size was reduced to 16, 14 and 11 Å, respectively. The surface area was also decreased. Jiang and co-workers used azide-appended building blocks to synthesize COFs and different alkynyl-combined groups were incorporated into the wall [214]. Thus, the pore size was precisely adjusted from 1.2 to 3.0 nm for COF-5 and 0.75 to 2.2 nm for NiPc-COF. Zhao and co-workers designed dual- and tripe-pore COFs, which made it possible to carry different guests at same time [215,216,217,218,219,220,221] and even be used for volatile iodine uptake [222]. What’s more, other researchers, such as Yaghi, Jiang and others, also reported heterogeneous pore structures [60,98,223,224,225]. Recently, Wang and co-workers suggested that, the pore shapes of COFs could be changed with different monomer concentration, including rhombus, parallelogram and Kagome morphologies [226].

Pore volume is another important factor for loading capacity of carriers. As for a certain COFs, their pore sizes and structures are constant but their pore volumes can be changed by managing interlayer interactions [208,227].

### 2.4. Morphological Control

In general, the synthesized COFs are irregularly shaped bulks, which limit their serviceable ranges especially in vivo application. However, COFs are hard to be reshaped, as they are insoluble in various solvents and also cannot be melted under high temperature [68]. At one time, it is a tough problem to prepare COFs in regular shape, especially in nanoscale. Fortunately, with time goes on, researchers have developed several methods to control the morphologies of COFs.

#### 2.4.1. 2D COF Films

The most studied regular morphology of COFs is nanofilm. Normally, it can be divided into two strategies, namely bottom-up and up-bottom methods. Bottom-up method to prepare COFs at the interface of solid-liquid [148], solid-vapor [180] liquid-liquid [190] and liquid-air [189] has been summarized in Section 2.2.5. By these approaches, nanofilms can be prepared at the surface of arbitrary substrates [174] and even in free-standing forms [190,192]. Up-bottom way, represented by exfoliation method, is a traditional method and inspired by the preparation of monolayer graphene sheet [228,229]. It can be executed by ultrasonic [64,230], immersion [231] and grind [120].

Zamora and co-workers suspended COF-8 powders in dry dichloromethane and sonicated them for 15 min under the frequency of 24 kHz [230]. After centrifuged, the synthesized COF films were suspended in the supernatant liquor with 10–25 layers. Banerjee and co-workers used ultrasonic exfoliation method to prepare thin layered covalent organic nanosheets (CONs) [232]. Surprisingly, theses CONs showed fast and high selectivity for the detection of nitroaromatic analytes, e.g., TNT. Zhao and co-workers proved that the exfoliated COF films could be blended with commercial polymers membranes and selectively adsorb CO_2_ [233]. Interestingly, Tsuru and co-workers found that exfoliated COF-1 nanosheets could be reassembled to form homogeneous membranes [234]. Dichtel and co-workers developed a gentle way to prepare hydrazone-linked COF films by immersed COF-43 into dioxane, H_2_O and DMF [231]. Banerjee and co-workers designed guanidinium-based ionic covalent organic nanosheets (iCONs), which possessed self-exfoliated properties due to the incorporation of ions [235]. What’s more, they also synthesized imine-based multilayered CONs by grinding [120].

The low yield of COF films is still a major problem remained to be solved. Up-bottom method is easy to handle and suitable to most of COFs but the morphology of products is hard to control. Bottom-up method can make up for this defect and shows more promising perspective.

#### 2.4.2. Other Regular Morphologies

The first class of regular bulk COFs was reported by Jiang and co-workers. In 2008, they built belt-shaped pyrene-based COFs, TP-COFs [236]. And later, they found porphyrin-based COFs, ZnP-COF, were uniform cubes and would be changed to disk-like or irregular shapes with different ratio of component solvent [152]. But the formation mechanism was still unclear.

**Figure 8:**
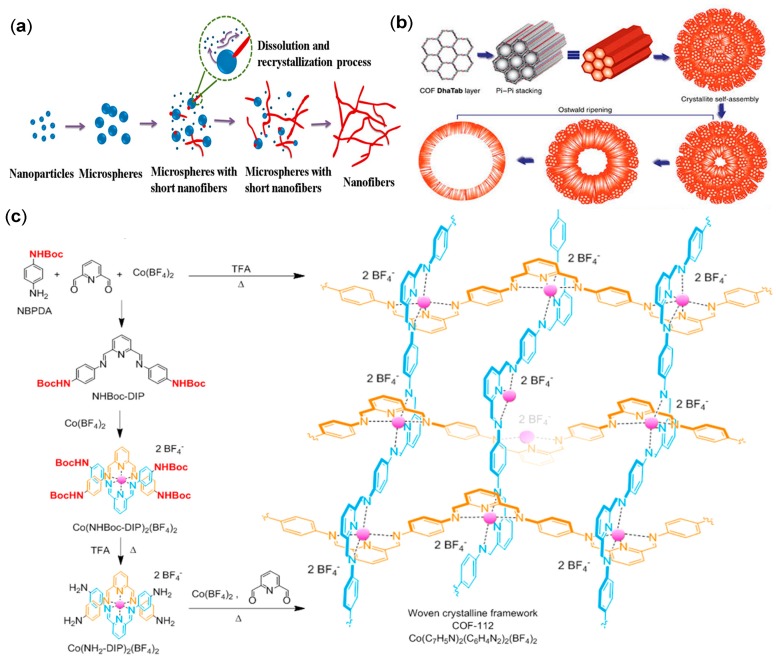
Schematic representations of the formation of (**a**) nanofibers, reproduced with permission from [237]. Copyright American Chemical Society, 2013; (**b**) hollow spheres, reproduced with permission from [116]. Copyright Springer Nature, 2015; (**c**) weaving COFs, reproduced with permission from [202]. Copyright American Chemical Society, 2017.

Liu and co-workers put forward a dissolution-recrystallization growth mechanism based on the dynamic imine bonds, as shown in Figure 8a [237]. SEM images showed that, the precursors formed irregular nanoparticles at first and then were aggregated into microspheres. When the temperature was increased to 180 °C, some of microspheres were dissolved and recrystallized onto the others and grew along one-dimensional directions. COF nanofibers were formed at last. After that, they used vapor-assisted solid-state approach to prepare COF nanofibers, too [154]. Based on this mechanism, they also obtained micro-octahedral COFs and provided a potential evolution process [238]. After the polycondensation of monomers, covalent organic nanosheets were formed and stacked quickly to form nanoplates because of interlayer π-π stacking. And then, these nanoplates were aggregated into octahedral morphology with rough surface. After that, the surface turned smooth gradually due to the reversible dynamic chemical properties. What’s more, they also studied the influence of organic acid, reaction time and stoichiometric ratio. All conclusions were supported by SEM observation and WAXS measurements. Similarly, Banerjee and co-workers constructed flower-like COFs and the sheet-like structures were also prepared by using π-π stacking of COF layers [99]. They also synthesized hollow spherical COFs (COF-DhaTab) through an Ostwald ripening process, as shown in Figure 8b [116,239]. The formed COF-DhaTab showed rod-shaped morphology at first and then were aggregated. The aggregations were gradually changed into spheres accompanying inside-out Ostwald ripening. At last, hollow spherical frameworks with a smooth surface were prepared. Through photochemical synthesis, COF-5 was even constructed in sea-urchin-like morphology [150]. The researchers suggested that, UV light made a different growth rate between the out-of-plane direction and in-plane direction. What’s more, Yaghi and co-workers constructed weaving COFs with assisted with copper (I) ions, as shown in Figure 8c [240].

COFs are also can be synthesized as uniform nanoparticles. When the amine building blocks were protected by *tert*-butyloxycarbonyl (Boc) groups, COFs were synthesized in homogeneously. Based on this result, Yaghi and co-workers used Boc-protected amine to build crystalline woven COF-112 which was around 200 nm [202]. Besides that, uniform LZU-1 nanocrystals were formed with PVP as the capping agent. The size of nanocrystals could be changed from 100 to 500 nm by adjusting the concentration of PVP. Dichtel and co-workers handled homogeneous synthesis through changing the components of reaction solvent [241]. They added acetonitrile into the traditional mixture solvents of dioxane/mesitylene to improve the solubility of monomers. With this approach, COF-5 nanoparticles would be formed as stable colloids. And the size would be decreased from 150 to 10 nm with the increase of acetonitrile concentrations. Before this study, they synthesized discrete macrocycles by modified HHTP monomers with incorporated hexa (ethylene glycol) monomethyl ether groups [242]. Yan and co-workers prepared spherical COFs with a diameter of 500 nm via room-temperature solution-phase synthesis [243]. Nano-sized COFs also can be prepared by template method. Growing imine COFs on the surface of MOF nanocrystals or Fe_3_O_4_ can fabricate hybrid nanoparticles no more than 200 nm [193,194,195]. Jiang and co-workers coated COFs on the surface of polymer spheres and the size was around 1 μm, which was decreased to 600–830 nm through pyrolysis [244].

To summarize, some morphologies of COFs have been reported though a few of them has been unraveled their formation mechanism. It is important to uncover the secret of COF formation and design more desired nanostructures.

### 2.5. Functional Modification

Modifiability is one of the most fascinating characters of COFs, which can improve their stability, adjust their structural characters and feature COFs with some special functions. A recent study from Smaldone and co-workers concluded that even small structural differences in building blocks have a tremendous effect on the crystallinity, surface area, pore structure and luminescence properties of COFs [245]. So that, modification is an effective way to expand the application scope of COFs and make them suitable for biomedical applications, which can be achieved by functionalized skeleton [236,246,247], incorporated substituents [213,248,249] and doped inorganic ions or particles [54,240,250,251].

#### 2.5.1. Functionalized Skeleton

Since COFs are consisted of building blocks, it is a straightforward way to functionalize COFs through changing their skeletons, which often makes COFs possess excellent photoelectric property. 

The first luminescent COFs was reported by Jiang and co-workers in 2008 [236]. They incorporated pyrene into the skeleton of boronate ester-linked TP-COF [236] and boroxine-linked PPy-COF [252]. These COFs showed highly blue luminescent due to the excitation of stacked pyrenes. After that, they used 1,3,6,8-tetrakis(4-formyphenyl)pyrene and hydrazine build azine-linked Py-Azine COF, which could emit a yellow luminescence and be used as a chemosensing detector [101]. Wang and co-workers also synthesized a 3D pyrene-based COF (3D-Py-COF) with a yellow-green luminescence, which may be used for explosive detection [253].

Porphyrin and their derivatives have proven to be intriguing functional building blocks for COF due to their unique photophysical properties, structural robustness, rich metal coordination chemistry and redox exchange behaviors [254]. Yaghi and co-workers reported imine and boronate ester linked porphyrin-based COFs with electrical conductivity and high charge mobility [246]. Jiang and co-workers found that porphyrin COFs exhibited high-rate hole, electron and ambipolar conducting properties by using different central metals [255]. Photosensitive porphyrin-based COFs were designed to generate singlet oxygen and used for photodynamic therapy [195,256].

Much similar to porphyrins, phthalocyanines are also square planar 18π aromatic macrocycles [257] and work as functional units. Jiang and co-workers integrated metallophthalocyanines into 2D COFs (NiPc COF), which exhibited a panchromatic light response especially for visible and near-infrared photons [151]. Later, they found central metal species could affect the π-electron density of phthalocyanines and play an important role in controlling the photoelectric [258]. Combined phthalocyanines with porphyrins, a novel C4 + C4 type 2D COFs were designed and used as light-harvesting antennae [259,260]. As electron donors, phthalocyanines can form donor-acceptor COFs with naphthalene diimides (NDI) as acceptors [247,261]. These COFs exhibited exceptional long-distance charge delocalization, long-lived charge separation and long-term charge retention. Besides, donor-acceptor COFs can be constructed with different electron acceptors such as naphthylenediamine [212,262], benzene-1,2,4,5-tetracarboxylic diimide [212], benzothiadiazole [263] and even triazine units [264].

Azobenzene (AZO) is a widely studied photo-responsive unit, which can be performed *trans*-to-*cis* photoisomerization under irradiation and can be recovered after heating [265]. A series of AZO-contained COFs have been reported and some of them have exhibited excellent responsiveness [121,266,267,268]. Under UV light, the pore walls of AZO-COFs will be broken and their crystallinity will be decreased, which can be used for controlled loading and release of guest monomers [266,267,268]. Another photo-responsive smart COF was designed with anthracene units [269]. Upon photoirradiation, cycloaddition reactions will take place among interlayer anthracene units, causing structural transformations. When heated, the COF will be regenerated. Jiang and co-workers used tetraphenylethene cored boronic acids (TPEBA) and 1,2,4,5-tetrahydroxybenzene (THB) to produce a kind of highly emissive COFs based on aggregation-induced emission (AIE) mechanism, which could be used for biosensing and bioimaging [224].

Efforts are also devoted in the increase of COFs’ conductivities. Incorporation of tetrathiafulvalene can provide COFs with tunable electrical conductivity [174,270,271]. Hydroquinone, bicarbazole, oligothiophene and pyridine units based COFs displayed high specific capacitance due to redox-active reactions [272,273,274,275,276]. Tetracyanoquinodimethane-derived CTFs were made for supercapacitors with high nitrogen content and large specific surface area [277]. Semiconducting COFs can be prepared by introducing bisaldehyde bdta units [189]. Many of the above-mentioned COFs, such as TP-COF [236] and NiPc COF [151], also have semi conductive property, too. Recently, Feng and co-workers synthesized a new anionic COF with γ-cyclodextrin (γ-CD) and tetrakis(spiroborate) tetrahedral [278]. γ-CD worked as the soft struts endow these CD-COFs with a prominent Li-ion conductivity. Besides that, the adsorption capability of CO_2_ was tunable with different counter cations.

In addition, Banerjee and co-workers used guanidinium halide as building blocks to make COFs possess self-exfoliated and antibacterial property [235]. Wang and co-workers introduced Salen units into imine COFs to prepare a new Salen-functionalized material with improved stability [118]. Based on the inherent Lewis acid-base interaction, Yaghi and co-workers used boronate ester-linked COFs to repeatedly absorb and release ammonia [279]. Jiang and co-workers synthesized a new benzimidazolium derivative to build ionic COFs, which efficiently absorbed CO_2_ and anionic pollutants [280]. Moreover, triarylamine and salicylideneanilines units were also used for selective adsorption [281,282].

#### 2.5.2. Incorporated Substituents

As we have mentioned earlier, incorporation of alkyl groups will tailor the pore size [213] and improve the hydrolytic resistance of COFs [113], while Banerjee and co-workers introduce hydroxyl into imine-based COFs and can improve their chemical stability by hydrogen bonding interactions [115] or enol-toketo tautomerism [99]. Besides that, there are still many other methods to incorporate substituents.

**Figure 9:**
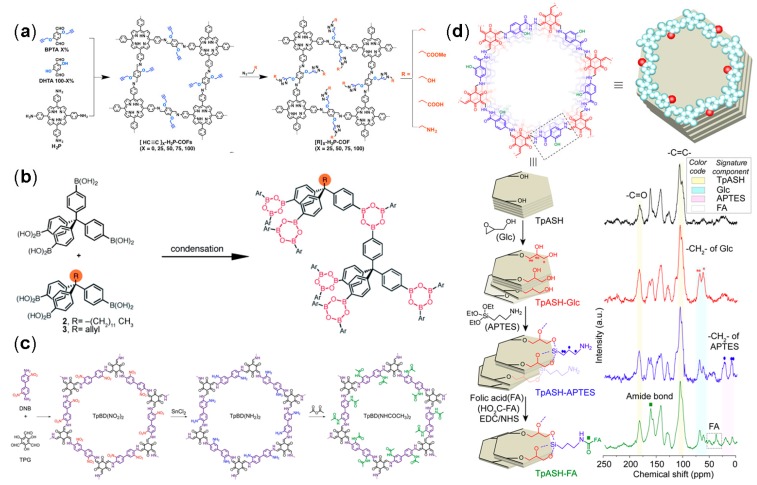
Schematic representations of the method to incorporate substituents: (**a**) azide-alkyne cycloaddition, reproduced with permission from [249]. Copyright American Chemical Society, 2015; (**b**) truncated mixed linker approach, reproduced with permission from [287]. Copyright John Wiley and Sons, 2012; (**c**) two-step post-synthetic method, reproduced with permission from [288]. Copyright American Chemical Society, 2016; (**d**) three-step post-synthetic method, reproduced with permission from [64]. Copyright American Chemical Society, 2017.

One of the most studied methods is through azide-alkyne cycloaddition, which is known as a classical post-synthetic approach [214,249]. As shown in Figure 9a, Jiang and co-workers introduced azide groups to 1,4-benzenediboronic acid and synthesized a novel azide-appended benzenediboronic acid (N_3_-BDBA), which worked as blocks to build COFs with HHTP [214]. After that, a series of alkynes contained groups can be used to modify the pore wall by click reaction. Similarly, they also prepared ethynyl-appended imine COFs and modified them with different azide compounds [249]. This method provides a practical way not only to tailor pores for selective adsorption but also endow COFs with some special functions. For instance, TEMPO units, a kind of redox-active species, were immobilized on the pore wall to make the COF an outstanding platform for energy storage [283]. And electron-accepting buckyballs (C_60_) were linked with phthalocyanine COFs to form donor-acceptor heterojunctions [284]. Pyrrolidine derivatives are widely used as catalysts for Michael addition reactions and will perform high stereoselectivity when connected with bulky substituents [285]. Thus, Jiang and co-workers introduced pyrrolidine units into COFs by azide-alkyne cycloaddition for chiral catalysis [119,248]. Besides click reaction, Yan and co-workers applied esterification reaction to incorporate chiral (+)-diacetyl-l-tartaric anhydride ((+)-Ac-L-Ta) on COFs, which was used for chiral separation [58]. Zhao and co-workers found that, when introduced into 2D COFs, the chiral units had a significant influence on their interlayer stacking [220]. Cui and co-workers designed different chiral pyrrolidine and imidazolidine groups and then modified COFs to control crystallinity and stability for asymmetric catalysis [286].

Dichtel and co-workers developed a general method to functionalize 3D COFs, named as truncated mixed linker (TML) approach [287,289]. It was well known that, COF-102, one of boroxine-linked 3D COFs, was condensed by a tetrahedral building block with four arylboronic acid moieties, as shown in Figure 9b [50]. In this approach, one of the boronic acid components was replaced with other functional groups, such as alkyl and allyl substituents [287,289]. And then, the resulting trigonal tris(boronic acid) was co-condensed with the former tetrahedral monomer to construct functionalized COF-102. Further modification could be achieved by a thiol-ene reaction for allyl-replaced COF-102 [289]. Similarly, Bein and co-workers used 4-mercaptobenzoic acid or 4-carboxyphenylboronic acid instead of partial benzene-1,4-diboronic acid to build functional COF-5 [290]. Ma and co-workers prepared thiol-modified COFs by thiol-ene reaction to remove mercury ions from solutions and the air [291]. When incorporated thioether groups, COFs can be used to selectively detect and remove mercuric ions [292,293]. Mancheño and coworkers introduced thiol groups into imine-based COFs and the resulting porous materials exhibited the highest retention value of Hg(II) among the reported works [294].

A two-step post-synthetic method has been developed to obtain amide functionalized β-ketoenamine COFs [288]. At first, nitro-contained benzidine was used to prepare COFs, as reported by Banerjee and co-workers [120]. And then, the nitro was reduced to primary amine. Furthermore, acetic anhydride was used to change primary amine to amide group, as shown in Figure 9c.

In addition, Banerjee and co-workers used cycloaddition reactions modified anthracene-containing COFs with *N*-hexylmaleimide molecules, which would disturb the π-π stacking interactions and result in the exfoliation of individual layers [179]. By this way, they prepared free-standing covalent organic nanosheets (CONs) at air-water interfaces. Recently, their groups have succeeded in anchoring folic acid onto other CONs by a three-step postsynthetic modification process [64]. The first step was to convert the phenolic hydroxyl groups of COFs to alkyl hydroxyl groups by glycidol. And then, 3-aminopropyltriethoxysilane (APTES) was used to incorporate amine group (–NH_2_) with CONs. At last, folic acid was conjugated to CONs with the EDC/NHS coupling reaction, as shown in Figure 9d.

Smaldone and coworkers incorporated fluorine into pore walls and improved the crystallinity, surface areas and porosity of these COFs, compared to the non-fluorinated analogue [295,296]. They explained that the fluorinated substituent stabilized the aromatic stacking interactions.

#### 2.5.3. Doped Inorganic Ions or Particles

Apart from covalent bond, coordinate bond and physical interaction are also used to modify COFs by doping inorganic ions or particles.

The first metal-nanoparticle-hybrid COFs were reported by Smit and co-workers in 2009. They doped Li atoms into 3D COFs to improve the capacity of hydrogen adsorption and storage [250]. Goddard and co-workers proved that, besides Li, other alkali metals, such as Na and K, also featured COFs with outstanding H_2_ uptakes [297]. What’s more, spiroborate-linked ionic COFs would have excellent absorption of H_2_ and CH_4_ if counter-cations were lithium ions [94].

Hybrid COFs are widely studied in catalysis. Wang and co-workers used the nitrogen atoms of imine-bonds to incorporate palladium acetate into COF-LZU1 and the resulting COFs showed excellent catalytic activity for the Suzuki-Miyaura coupling reaction [54]. Similarly, Jiang and co-workers used phenolic hydroxyl groups to combine VO(acac)_2_ [298] and Zhang and co-workers applied acylhydrazone bond to connect MoO_2_(acac)_2_ [299]. Yaghi, Cheng and co-workers used cobalt porphyrin to prepare COF-366-Co and COF-367-Co, which can effectively reduce CO_2_ to CO by electrocatalysis in water [53]. Co(II) ions doped into bipyridine-containing COFs made the COFs as an OER catalyst in a neutral pH buffer solution [300]. Triazine units were used to anchor Ru atom for selective alcohol oxidation [301]. These are also many other works about COFs doped by metal ions or atoms for catalysis [302], which will contribute to the development of catalysis.

More than a coordinate bond, Banerjee and co-workers directly immobilized Au and Pd nanoparticles into COFs by in situ reduction [122,303]. This composite catalyst showed high activity for heterogeneous catalysis. Their groups also deposited CdS nanoparticles onto the surface of 2D COF matrix and the hybrid sheets worked as photocatalysts for H_2_ production [304]. Similarly, chloro(pyridine)cobaloxime, Co, Ni and Pt particles were introduced into COFs for catalysis [305,306,307,308].

Dopants can impact the conductivity of COFs. Sulfur was impregnated in COFs, e.g., CTF-1 [251], Azo-COF [309] and COF-1 [310], which can be used for cathode of lithium-sulfur batteries. Electropolymerization of the 3,4-ethylenedioxythiophene (EDOT) method was used to enhance the electrical conductivity of 2,6-diaminoanthraquinone-containing COF films and the resulting films possessed stable capacitances for at least 10,000 cycles [311,312]. The proton conduction ability of COFs could be increased by incorporating phytic acid, phosphoric acid, acetic acid, LiCl and PW_12_O_40_^3−^ [166,313,314,315]. Incorporation of metal ions has a notable effect on the morphologies of COFs. Yaghi and co-workers designed 3D woven COF-505 with copper (I) ions [240] and COF-112 with cobalt (II) ions [202]. Jiang and co-workers found that, the guest molecules were involved in the crystallization process and the crystallinity could be adjusted by designing suitable guests [316].

### 2.6. Properties for Biomedical Applications

Along with those great progresses, COFs possess multiple prominent advantages that allow these materials for theranostic applications.

(1) Dynamic covalent linkages for stimuli-responsibility and biodegradation

Instead of traditional covalent bonds, coordinate bonds and physical interactions, COFs utilize dynamic covalent bonds as linkages, which can keep their structure in normal conditions and be broken by stimulations such as acid [317]. These characters make COFs be stable enough to preserve their structure before reaching the target tissues and biodegrade after finishing their task. Moreover, they can rapidly release cargos under stimulation, especially for imine and its analogue linkages frameworks. Polymer-covered boronate ester and spiroborate linked COFs also can be promising candidates for drug delivery, as the reversibility and dynamic nature of the boronate ester offered not only pH and glucose dual-responsive features but also adaptive mechanical behaviors, such as shear-thinning property for injection, [318,319,320]. Recently, CTFs were found to possess pH responsiveness due to the protonation and deprotonation of triazine units [131]. Apparently, the stimuli responsiveness and biodegradation make COFs a kind of ideal carriers for therapy.

(2) High surface area and large pore volume for high loading capacity

Due to their unique framework structures, COFs possess high surface areas and pore volume. For example, the BET surface area of COF-102 is 3472 m^2^/g and COF-103 is 4210 m^2^/g; the pore volume of COF-103 is 1.66 m^3^/g and COF-10 is 1.44 m^3^/g [50,207]. These features are much better than most of porous materials, such as mesoporous silica and carbons [31,32]. What’s more, COFs are constructed by aromatic compounds and can be used to load aromatic monomers through π-π stacking. Thus, COFs possess high loading capacity of drugs and biological molecules.

(3) Tunable pore structure for controlled release

As described in Section 2.3, the pore sizes and shapes of COFs can be adjusted through changing building blocks or post-modification. Since pore sizes and shapes have an effect on the diffusion of guest monomers, COFs with tunable pore structures can be used to carry different pharmaceutical agents and control their releasing rate.

(4) Excellent photoelectric properties for biosensing and bioimaging

Mostly, COFs contain π-conjugated system and laminated structures, which feature them with excellent photoelectric properties. For instance, polycyclic aromatics units (e.g., tetraphenylethenes and pyrenes) can make COFs emit fluorescence and electron donors or acceptors units (e.g., naphthalimides, porphyrins and phthalocyanines) can endow COFs with proton conductivity. Based on those properties, COFs show a great potential in biosensing and bioimaging.

(5) Unique tailorable attributes for desired performance

Since COFs consist of building blocks with high tailorability, extensive materials will be designed as building blocks and introduced into their skeletons. By this way, COFs can exhibit various desired performance, such as produce singlet oxygen for photodynamic therapy [256]. What’s more, tailored attributes of COFs pave a way for the study of structure-activity relationship.

(6) Outstanding modifiability for versatile functionalities

Modification is an effective way to enhance the applicability of COFs. Inspired by other nanoparticles, surface modification can improve their biocompatibility and targeting ability [41,321]. Since MOFs can regulate drug release profiles by incorporating different substituents on the pore wall [322], COFs may achieve similar results. Biological probe and drugs even can be linked onto COFs by post-synthesis method. With proper ligands, inorganic pharmaceutical agents (e.g., cisplatin) may be connected on COFs. There are also multifarious possible functionalities waiting for further exploration. These properties open the door for the applications of COFs in biomedicine.

## 3. COFs for Biomedical Applications

Due to their great potentials and unique features in diagnosis and treatment, COFs have been explored in drug delivery, photothermal therapy, photodynamic therapy, biosensing, bioimaging and other biomedical applications. Even though in their early stages, COFs have attracted intensive attention of researchers and a surge of studies have been reported in the past several years.

### 3.1. Drug Delivery

The early example of applying COFs to drug delivery was reported by Yan and co-workers [63], who designed two new 3D polyimide COFs with different pore size, named PI-COF-4 (13 Å) and PI-COF-5 (10 Å). Ibuprofen (IBU, molecular size 5 Å × 10 Å) was chosen as a model drug, as it can be entrapped by the pores of PI-COFs. TGA analysis proved that the drug loading efficacy (DLE) was as much as 24% (PI-COF-4) and 20% (PI-COF-5), respectively. And drug release was sustained for more than 6 days. Besides IBU, PI-COFs were also suitable to deliver captopril and caffeine, achieving similar results with that of IBU.

Porphyrin-based covalent triazine frameworks (PCTFs) were also used in drug delivery [132]. The authors believed that, the acid group decorated in IBU could interact with the triazine rings of COFs, which would have a positive effect on drug loading and controlled release. TGA experiment indicated that the DLE was about 19% for metal-free PCTFs and 23% for Mn-containing PCTFs. For both PCTFs, the total release amount was beyond 90% within 48 h. These results are no better than those of abovementioned PI-COFs. It may be because PCTFs had irregular morphologies and wider pore size distribution due to the poor crystallinity of triazine-based COFs [133].

**Figure 10:**
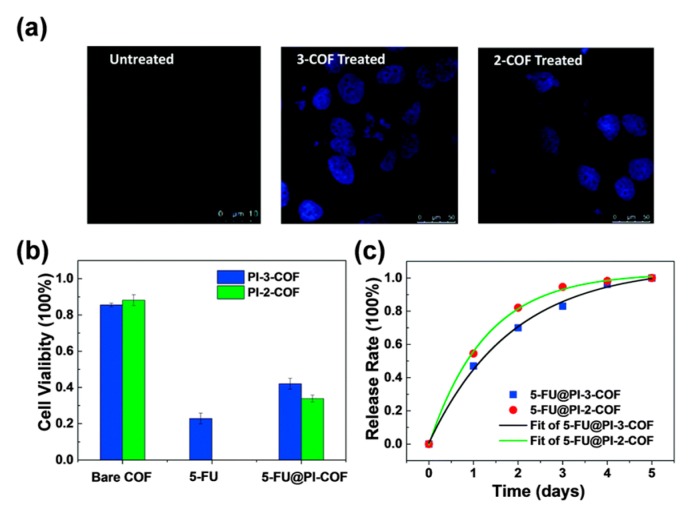
(**a**) Confocal images for cell uptake; (**b**) Quantitative MTT analysis for cell viability; (**c**) Release profiles of drug loaded COFs. Reproduced with permission from [323]. Copyright Royal Society of Chemistry, 2016.

Although a number of studies were carried out to investigate in vitro drug release from various COFs, few works are focused on the in vivo biocompatibility and cytotoxicity. Zhao and co-workers conducted cell experiments with two imine-linked 2D COF, PI-2-COF and PI-3-COF [323]. Both two COFs showed high drug loading capacity for 5-FU, captopril and IBU and even reaching to 30 wt %. The release rates of 5-FU were similar for both PI-n-COFs and most drugs were released out within 3 days (Figure 10c). More importantly, these COFs were uniform spherical nanoparticles with a diameter of 50 nm, when dispersed in PBS solution with a bit DMSO as an additive. This feature made carriers in a suitable size for cell uptake and in vivo drug delivery. Confocal images proved the effective uptake of drug loaded nanoparticles by the cells MCF-7 (Figure 10a). Quantitative MTT analysis indicated that, two COFs both exhibited good biocompatibility. But the viability of cells was decreased to 10% when treated with 5-FU loaded hybrids for 48 h, as shown in Figure 10b. Lotsch and co-workers designed a new imine-based TTI-COF to deliver Quercetin [324]. TTI-COF could keep its morphology in water and weak acids for more than two days. Molecular dynamics simulations proved that the Quercetin was bound on the pore wall by C-H-π and H-bond interactions. For this reason, TTI-COF seemed to be able to carry more drugs. However, TGA analysis was failed to calculate the accurate drug-loading rate, since Quercetin was decomposed slowly over a wide temperature range. What’s worse, in vitro drug-release study was also not successful due to the fact that the Quercetin was hard to dissolve in PBS and easy to oxidize [325]. Cell experiments indicated that the drug-loaded COF successfully killed most of human breast carcinoma MDA-MB-231 cells while the bare COFs showed no cytotoxicity. In spite of some inadequacies, this work carefully discussed every detail from material preparation to cell experiment and showed us valuable experiences to design this new carrier.

Stimuli-responsibility is one of most fascinating properties to achieve real-time monitoring and on-demand drug release [317,326]. Beside the inherent acid sensitivity of boronate and imine based COFs, researchers have endowed COFs with more environment responsive features to make the drug delivery and release in a controllable way. Huh and co-workers prepared pH sensitive CTF nanoparticles (NCTPs) through the Friedel-Crafts reaction [131]. DOX was loaded in NCTP as model drug by hydrophobic and π-π interactions. When pH decreased to 4.8, the triazine units became protonated and the hydrophobic interactions between DOX and NCTP were weakened. Thus, release rate was faster than that under pH 7.4 PBS. More interestingly, the COFs as drug carriers can provide more flexibility and additional option for access to controllable drug release, due to their unique photoelectric properties. For example, Lei and co-workers applied self-condensation of 4,4′-phenylazobenzoyl diboronic acid (ABBA) to build single-layer photoresponsive COFs on the surface of HOPG [266]. Under UV irradiation, the frameworks would be destroyed and release the gust, copper phthalocyanine (CuPc). Moreover, the destruction of COFs could be recovered through annealing. This finding will offer a possible on/off switch for drug release.

**Figure 11:**
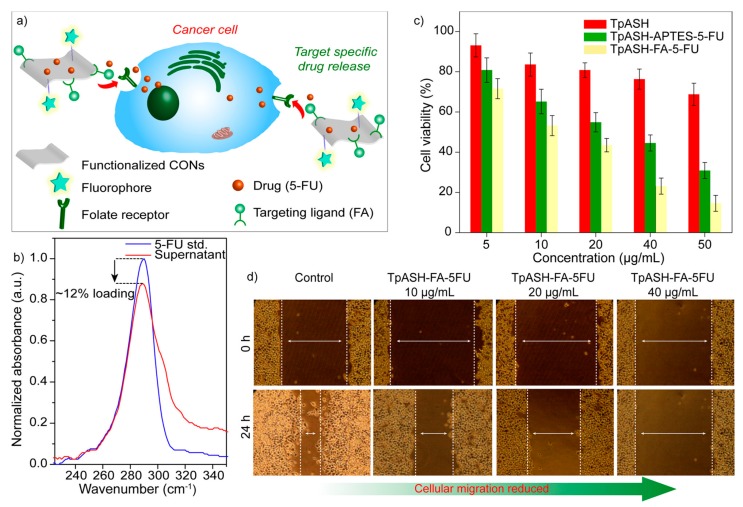
(**a**) Schematic representation of targeted drug delivery by CONs (sheet-like material denotes CONs here); (**b**) drug loading study of 5-FU by UV−vis; (**c**) MTT assay on MDA-MB-231 cell lines showing cellular viability, and (**d**) Comparison of cellular migration study between control and TpASH-FA-5-FU treated sets. Reproduced with permission from [64]. Copyright American Chemical Society, 2017.

Banerjee and co-workers did a series of exploration about imine based COF for drug loading and release [64,116,198]. In 2015, they synthesized hollow spherical covalent organic framework through template-free method [116]. The drug loading capacity was only 0.35 mg/g for DOX calculated by UV-Vis absorbance spectra. The release rate was very slow and more than 50% was remaining after 7 days in pH 5 phosphate buffer. After that, they designed self-standing porous COF membranes to sieve larger molecules (more than 1 nm) such as rose bengal, tetracycline, curcumin and so on [198]. Since EPR effect is not as effective as expected and only about 5% of the administered nanoparticles can reach the target tumor [42,327], modified with target groups is a possible way to improve the use of nanocarriers. Banerjee and co-workers took the first step in the COFs for targeted drug delivery (Figure 11). They modified covalent organic nanosheets with folic acid (named as TpASH-FA) through the postsynthetic modification method (as referred in Section 2.5.2) and 5-FU was chosen as model drug [64]. Although the loading efficiency of these nanosheets (only 12%) was lower than many other COFs [63,131,132,323], they exhibited pretty good anticancer activity and only 14% of MDA-MB-231 cells survived at a dosage of 50 μg/mL.

**Figure 12:**
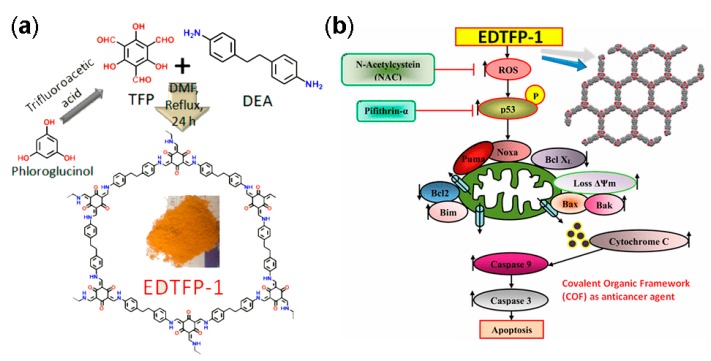
Schematic representations of the preparation of EDTFP-1 (**a**) and its induced apoptotic pathway (**b**). Reproduced with permission from [328]. Copyright American Chemical Society, 2017.

Apart from carrying anti-cancer drugs, functional COFs can kill tumor cells by themselves. Bhaumik and co-workers reported an interesting work in which phloroglucinol contained COFs were used as anticancer agents [328]. They applied 2,4,6-triformylphloroglucinol and 4,4’-ethylenedianiline to construct nanofiber like COFs (EDTFP-1) with a diameter of 22–30 nm (as shown in Figure 12a). The borne phloroglucinol derivative could accelerate ROS generation and caused the apoptosis of cancer cells [329]. Results of cell experiments demonstrated that EDTFP-1 successfully induced mitochondrial dependence apoptosis associated with DNA fragmentation, mitochondrial membrane potential loss, phosphatidylserine externalization and pro- and antiapoptotic proteins unbalance (as shown in Figure 12b). Thus, EDTFP-1 displayed obvious cytotoxicity against cancer cells, like HCT 116, HepG2, A549 and MIA-Paca2. This finding will open a door for COFs to work as anticancer agents.

Herein, we summarized those works in Table 3. From the table, we can find that, almost drug-loading COFs are linked by C–N bonds, which are believed to be stable in water. Although COF_ABBA_ was linked by boroxine bonds, neither loading efficacy nor release rate was studied. The pore sizes are most designed to fit the model drug but the morphologies of these carriers are various instead of uniform nanoparticles, which need further exploration. Generally, the drug loading efficacy (DLE) of COFs was rather high and exhibited pretty well biocompatibility. Although COFs have shown their pharmaceutical potential, several limitations markedly hamper their clinical translation. The intricate structural characteristics demand careful engineering of the COFs in order to realize the desirable effect. The complexity of the COFs preparations caused a key issue needed to be addressed in the scale-up production and batch-to-batch reproducibility. Challenges still exist in terms of delivery of the cargo to the targeted site as well as efficient clearance of the COFs once they have finished their mission in vivo. Although several studies have tested the efficacy of COFs formulations and their safety, few studies on their long-term accumulation and degradation profiles were carried out. However, we believe that continuous improvements in the design and detailed investigations on their in vivo behaviors will help to take COFs as nanocarriers from bench to bedside.

### 3.2. Photothermal and Photodynamic Therapy

Besides chemotherapy, COFs also can be used for photothermal therapy (PTT) [193] and photodynamic therapy (PDT) [195,256].

**Figure 13:**
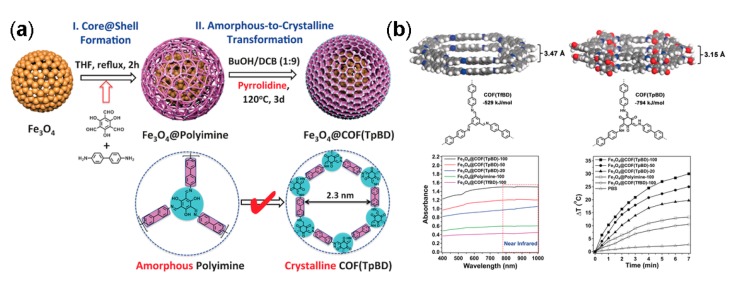
Schematic representations of preparation (**a**) and photothermal effect (**b**) of Fe_3_O_4_@COF. Reproduced with permission from [193]. Copyright John Wiley and Sons, 2016.

Inspired by conjugated microporous polymers [330], Guo and co-workers attempted to cover imine-linked COFs on the surface of Fe_3_O_4_ nanoclusters for PTT, as shown in Figure 13a [193]. The resulting Fe_3_O_4_@COF microspheres increased the system temperature by 25 °C (Figure 13b) and the photothermal conversion efficiency (21.5%) was comparable to some widely studied photosensitizers such as Au nanorods [14,331]. Moreover, the Fe_3_O_4_ core featured the microspheres with magnetic target characteristic.

**Figure 14:**
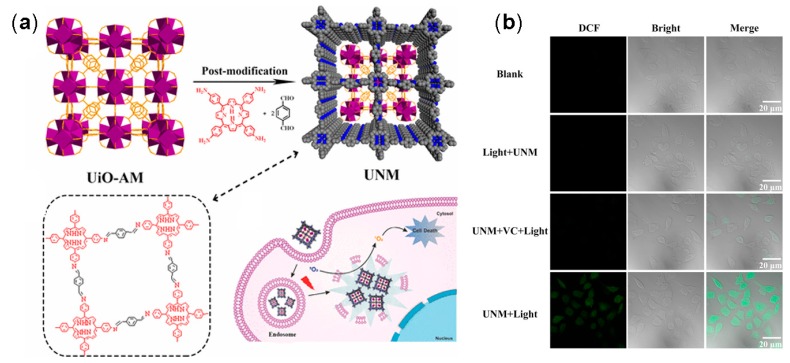
Schematic representations of preparation (**a**) and photodynamic effect (**b**) of MOF@COF. Reproduced with permission from [195]. Copyright American Chemical Society, 2017.

Porphyrin and its derivatives can generate singlet oxygen (^1^O_2_) under photoirradiation and widely studied as photosensitizers for PDT [12]. Taking porphyrin derivatives as building blocks to construct photosensitive COFs is a promising way to avoid the aggregation of porphyrin macrocycles in aqueous media and improve their efficiency in vivo. Wang and co-workers designed 3D porphyrin-based COFs, which possessed excellent photosensitive and could be used to produce reactive singlet oxygen under photoirradiation [256]. They also found that, the photosensitive properties could be tuned by the incorporation of metal ions into the porphyrin macrocycle. Xie and co-workers grew imine-linked COFs on the surface of amine decorated metal-organic framework (MOF) to prepare porphyrin-based COFs with uniform nanoscale structures, as shown in Figure 14a [195]. The amine modified MOF was regular octahedron around 165 nm. When covered with COFs, the hybrid particles (referred as UNM) were nearly spherical shapes with average size of 176 nm determined by DLS. Confocal laser scanning microscopy images showed that UNM could be endocytosed by HeLa cells. As shown in Figure 14b, with 2′,7′-dichlorodihydrofluorescein diacetate DCFH-DA as indicators, bright green fluorescence could be observed under light irradiation, which was contributed to the production of singlet oxygen. MTT assay proved that UNM had significant cytotoxicity for both HepG2 and HeLa cells under irradiation but nearly no cytotoxicity can be found without light.

### 3.3. Biosensing and Bioimaging

The π-conjugated system and photoelectrical properties of COFs have been widely studied in catalysis [52,53], proton conduction [61,121] and energy storage [272,277]. Lately, these features are developed for biosensing and bioimaging [175,332,333].

**Figure 15:**
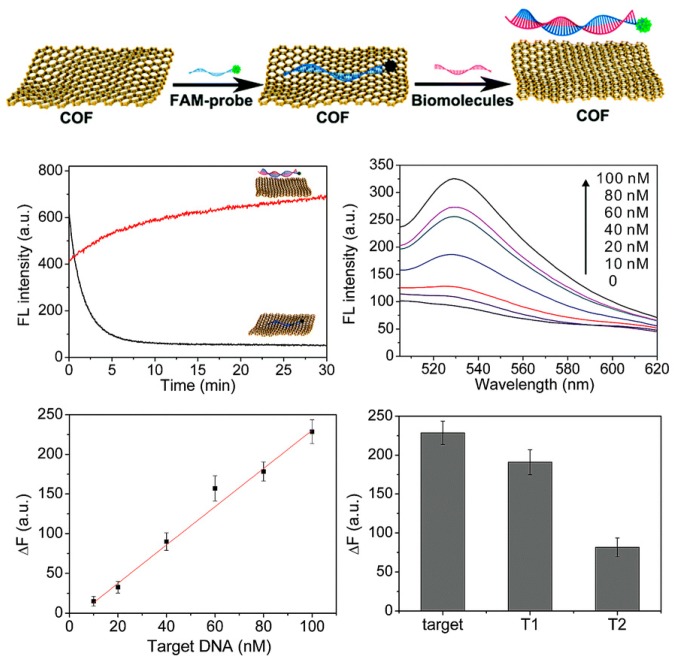
Schematic representations of COF-based sensing platform for DNA detection. Reproduced with permission from [332]. Copyright Royal Society of Chemistry, 2017.

One of biosensors was designed based on the electrochemical activity of COF films. Fang and co-workers synthesized imine-linked COF films onto amino functionalized silicon wafers and the presence of amino groups can endow the hybrid films with positive charge [175]. Negative charged biomolecules, e.g., BSA and probe DNA, would be adsorbed on the surface by electrostatic interactions to strengthen the electrochemical activity of COF films. Electrochemical impedance spectroscopy was used to detect this change. Thus, the biological signals were converted into electric signals. The other biosensing application is based on the π-conjugated system of COFs [332,333]. Utilizing the π-π stacking effect, fluorescent dye labeled DNA probes would be adsorbed on the surface of COF films and the fluorescence of dye was quenched via fluorescence resonance energy transfer (FRET) [333]. When the target DNA combined with probes, the interactions between DNA probes and COF films would be weaken and the fluorescence would be recovered. Similarly, carboxyfluorescein-labeled probe (FAM-probe) was adsorbed on the β-ketoenamine linked COFs by hydrogen bond and π-π stacking interactions [332]. As shown in Figure 15, when the target biomolecules had interaction with FAM-probe, the fluorescence will be enhanced. This new platform exhibited highly sensitive and selective DNA and adenosine 5′-triphosphate.

Beside biomolecules, COFs also can be applied to detection of monomers and metal ions. Jiang and co-workers designed azine-linked pyrene-based COFs, in which the azine units extended π delocalization over the 2D skeleton, making the framework able to emit a yellow luminescence [101]. Furthermore, azine units have lone pairs of electrons and provide docking sites for hydrogen-bonding interactions with guest molecules. Thus, these frameworks can be used to detect 2,4,6-trinitrophenol with high sensitivity and selectivity, since phenol units could form hydrogen-bond with azine units and nitro groups could cause the quenching of fluorescence. Similarly, another 3D pyrene-based COFs were also used for chemosensing [253]. Jiang and co-workers used AIE mechanism to prepare highly emissive boronate ester-linked COF materials [224]. Upon to ammonia, the luminescence of COFs was decreased because of the Lewis acid-base interaction between boronate ester and ammonia. This discovery will be used for ammonia detection at sub-ppm level. When thioether is incorporated onto the pore wall, COFs can be used to selective detection and removal of Hg^2+^. Wang and co-workers designed a thioether-functionalized COF, COF-LZU8, with strong fluorescence [292]. When upon Hg^2+^, the fluorescence was reduced due the coordination interaction between the thioether groups and Hg^2+^. More importantly, the fluorescence was recovered when sulphur ions were added to remove Hg^2+^. Yang and co-workers designed two kind of polyimide based COFs, which emitted strong fluorescence in solution and the fluorescence would be quenched by Fe^3+^ [334]. This property can be used for selective chemosensor of Fe^3+^.

Triazine-based frameworks NCTPs exhibited a strong emission property and can be used for bioimaging due to their extended π conjugation in the frameworks [131]. What’s more, AIE effect and blue luminescence of conjugated COFs are hopeful to be developed into bioimaging [224,236,252].

### 3.4. Other Biomedical Applications

**Figure 16:**
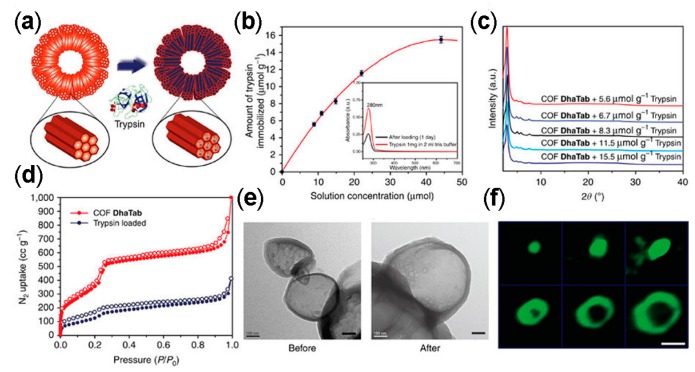
(**a**) Schematic representation of trypsin immobilization; (**b**) Amount of trypsin loading in COF-DhaTab; Evidence of trypsin immobilization through (**c**) PXRD patterns; (**d**) N_2_ adsorption isotherms; (**e**) TEM images; and (**f**) Confocal Z stacks. Reproduced with permission from [116]. Copyright Springer Nature, 2015.

Based on their selective adsorption ability, COFs can be applied in protein immobilization, biomolecular adsorption and hazardous substance removal. Immobilization of enzymes plays an important place in biomedical industries [335]. Banerjee and co-workers designed hollow spherical COFs (COF-DhaTab) for trypsin immobilization [116]. Although trypsin was globular protein with a diameter of 3.8 nm, which was a bit larger than the pore size of COF-DhaTab (3.7 nm), the enzyme adjusted its conformation to fit the pore size due to its soft characters. As shown in Figure 16, various methods were used to demonstrate the immobilization of trypsin into the COFs and the loading capacity was as high as 15.5 μmol/g. Cai and co-workers prepared core-shell structured Fe_3_O_4_@COFs by way of the room-temperature synthetic method [194]. Based on the hydrophobic effect, the composite nanoparticles absorbed hydrophobic peptides and repel hydrophilic peptides. With the assistance of magnetic responsiveness of Fe_3_O_4_ cores, hydrophobic peptides were separated from mixture system. What’s more, these particles were used for selective enrichment of peptides and exclusion of proteins. This feature has a great potential in real-world application, such as the analysis of human serum. When incorporated amine groups onto the pore walls, COFs were used for adsorption of lactic acid [288]. Other works showed that, when modified with sulfur derivatives, COFs were used to remove Hg^2+^, Hg^0^, Pd^2+^ and Cu^2+^ [291,293].

**Figure 17:**
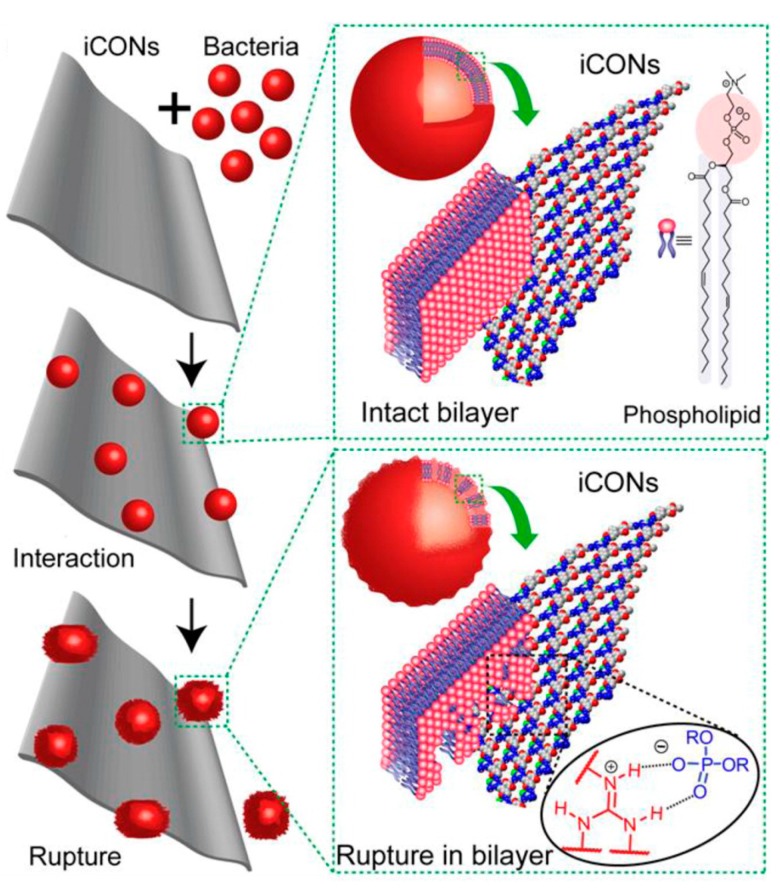
Schematic representations of iCONs for antimicrobial applications. Reproduced with permission from [235]. Copyright American Chemical Society, 2016.

COFs can be used for antimicrobial applications. Banerjee and co-workers used 1,3,5-triformylphloroglucinol and guanidinium halide to build self-exfoliated ionic covalent organic nanosheets (iCONs) [235]. Since guanidinium units can form hydrogen-bond with the oxoanions of phosphate, positively charged iCONs can break negatively charged phospholipid bilayer of bacteria, as shown in Figure 17. Antibacterial studies testified that these nanosheets exhibited excellent antimicrobial activity against both Gram-positive and Gram-negative bacteria. Furthermore, they mixed iCONs and polysulfone (PSF) to fabricate iCONs@PSF membrane as antimicrobial coatings. What’s more, COFs are expected to deliver bioactive gases, like nitric oxide (NO), as MOFs do [336].

## 4. Conclusions and Perspective

Covalent organic frameworks, a representative material of porous organic polymers, have been widely explored in various fields and play an important role in nanotechnology. In this review, we present the significant developments of COFs formed by dynamic linkages, synthetic method, pore design, morphological control and functional modification. With these achievements, COFs exhibit numerous notable advantages, such as stimuli responsiveness, biodegradability, high surface area, large pore volume, tunable pore structure, photoelectric properties, unique tailored attributes and outstanding modifiability, which pave a way for this new porous framework to be used for biomedical purposes. Most importantly, the complementary functional design of skeletons and high flexibility in the control of pore geometry may provide a powerful means of exploring COFs for challenging biomedicine issues. Especially for the unique photoelectric property, COFs are expected to provide an ideal theranostic platform due to the convenience and ability to integrate the drug therapy and photoelectric diagnostic function in one COFs-based system. As a result, it was no wonder, although at its fancy stage, that the applications of COFs in drug delivery, photothermal therapy, photodynamic therapy, biosensing, bioimaging and other possible diagnosis have significantly increased and received increasing attention with the development of new COFs materials. However, despite their many favorable characteristics and great progress, most of the currently available COFs face many challenges in terms of preparation complexity, regulated morphology, hydrolytic stability, targeted delivery, controlled release, long-term biocompatibility, which indeed impair their practical applications in biomedical and pharmaceutical fields. As a result, considerable efforts are urgently required to design and fabricate COFs with well-defined morphologies and desirable properties. Especially, future work need to focus the following issues.

(1) Scale-up preparation of COFs with controlled and uniform morphology

Despite the great progress made in the field of COFs design and preparations, none of the COFs and corresponding preparation methods are perfect. Firstly, as a class of crystalline and porous materials, the crystallinity and porosity of COFs are of central importance for many applications. Especially for pharmaceutical and biomedical applications, the uniformity in the materials properties and therapeutic performances and the reproducibility in the preparation process and product quality are two very important requirements. However, due to the complexity in the molecular structure of COFs and high heterogeneity in reaction environment, the biomedical applications of COFs not only face the almost inevitable crystal defects, stacking faults, pore dislocations and uncontrolled morphologies but also need to address the scalable production and batch-to-batch reproducibility. More importantly, through the existing approaches, it is still very hard to prepare stable COFs with a uniform size in the nano range. And thus, it is imperative to develop a simple and effective method to construct nanoscale COFs with precise crystal structure and well-defined pore geometry in a scalable process, achieving high uniformity, reproducibility and thus high-performance of the synthesized COFs. 

Secondly, the building blocks for constructing COFs are usually expensive or not commercially available. The most common starting materials are mainly a variety of polycyclic aromatic derivatives with multiple reactable groups, whose preparations generally involve multistep tedious reactions under drastic reaction conditions and complicated purification steps. Especially, these COFs materials and their degradation products always suffer from high toxicity, making them unsuitable for biomedical applications. The rational design and optimization of the building blocks in COFs have become a focus for research in the biomedical fields to enhance COF biosafety.

In addition, it was worth pointing out that, at present, there are still no effective tools to visualize and analyze the stacking and topological structures of COFs, further limiting the ability to control the quality and shape of COFs.

(2) Hydrolytic and hemodynamic stability

Owing to the reversible nature of the linkages in the COFs, these crystalline porous polymers often suffer from limited stability, especially in the humid, acidic and basic environments. On the one hand, these reversible linkages endow the COFs with desirable sensitivity for some special purposes, such as controlled drug release and environment-triggered degradation. One the other hand, the limited stability will markedly reduce the materials service-life time and thus significantly prevent their practical applications. As a result, the synthesis of a stable COFs that is robust against harsh conditions or physiological environments remains a major issue that need to be addressed. The design and development of new linkages for fabrication of COFs may be an effective solution to overcome these issues.

After injection or implantation in body, the interactions between the materials with serum proteins, cells and tissues will determine the performance of biomedical materials. Especially for the materials used in drug delivery, they should have the ability to avoid the clearance of the nanocarriers by the reticuloendothelial system and enable the circulation time to overcome the biological obstacles in the path of delivery. Therefore, to achieve high therapeutic efficacy and low side effects, the idea COFs for drug delivery should be able to sufficiently stabilize to tightly retain the drug in the bloodstream and maximize the accumulation in targeted site. However, so far few of COFs can simultaneously meet these requirements. Some of these issues may be effectively overcome by surface modification though inert hydrophilic polymers, such as PEG, or by embedding them in a supporting matrix or substrate, such as hydrogels or liposomes.

(3) Biocompatibility and systemic toxicity

A detailed and careful assessment of the factors that can influence the biocompatibility and toxicity of COFs is crucial for their safe and sustainable development in the pharmaceutical and biomedical fields. However, although there are several studies performed to test the cellular toxicity of COFs, as a novel kind of nanomaterials, the hemocompatibility, histocompatibility, cytotoxicity and neurotoxicity of COFs are unclear at this stage, let al.one the long term in vivo assessments on the absorption, distribution, metabolism and excretion of COFs. Especially considering polycyclic aromatic compounds as the starting materials of COFs, more studies of systematic toxicity, biocompatibility and biological effects, especially in animal models, are urgent and fundamental issues that require thorough investigation, before COFs-based biomaterials can be applied for human welfare. In this context, the exploration of new compatible building blocks and linkage reactions may be the key to expand the COFs biomedical applications.

Although there is still a long way before clinical applications of COFs, we believe the rapid development of these materials will make them become a potential platform for biomedical and pharmaceutical purposes, opening up some new avenues for exciting opportunities to improve human welfare. Undoubtedly, collaborative, multidisciplinary research efforts are still needed for more successful applications of COFs in biomedical science.

## Figures and Tables

**Table 1:** Previous reviews of covalent organic frameworks (COFs).

Categories		Main Contents
Comprehensive Reviews		Structure designs, synthetic methods, characterizations and applications [65,66,67,68,69]
Structures	Linkages	Different linkages [55,56,57]; New linkages beyond boron chemistry [70]; C–N based COFs [71]; Triazine-based COFs [72]; Schiff-based COFs [73]; imine-based COFs [74]
	Building Blocks	Porphyrin- and phthalocyanine-based COFs [75]; Boronic acid-based COFs [76]
	Pore Designs	Multiparous 2D COF [77]
	Architectures	Structure design [48,78,79]
Applications	Absorption & Storage	Energy storage [62,80,81]; Gas storage [55]; Hydrogen storage [82,83,84]; CO_2_ capture [56,57]
	Catalysis	Heterogeneous catalysis [72]; single-site catalysts [85]; Electrocatalysts [86]
	Analysis & Detection	Electronic and optical applications [62,87]; Analytical applications [88]; Chemical sensing [89]
	Comprehensiveness	Various applications [78,90]
Synthetic Method		Various synthetic methods [73]; Mechanochemical synthesis [91]

**Table 2:** Typical linkages for building COFs.

Bonds	Linkages	Characters	Reference
B–O	Boronate ester 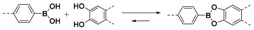	Crystallinity: Excellent; Thermostability: 600 °C; Chemical stability: Sensitive to water, acid, base, alcohols and atmospheric moisture.	[51]
	Boronxine 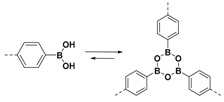	Crystallinity: Excellent; Thermostability: 500 °C; Chemical stability: Sensitive to water, acid, base, alcohols and atmospheric moisture.	[51]
	Borosilicate 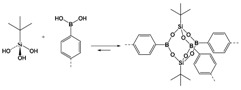	Crystallinity: Excellent; Thermostability: 450 °C; Chemical stability: 1 h in air.	[93]
	Spiroborate 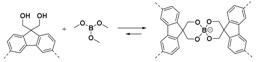	Crystallinity: Excellent; Thermostability: Loss 7–12% at 400 °C; Chemical stability: 2 d in water and 1 M LiOH; sensitive to acid.	[94]
C–N	Imine 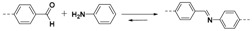	Crystallinity: Good; Thermostability: 500 °C; Chemical stability: Better than boron-based COFs.	[95]
	Hydrazone 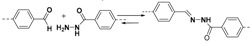	Crystallinity: Good; Thermostability: 280 °C; Chemical stability: Better than imine-linked COFs.	[96]
	Imide 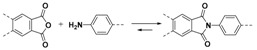	Crystallinity: Good; Thermostability: 530 °C; Chemical stability: Better than boron-based COFs.	[97]
	Amide 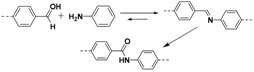	Crystallinity: Good; Thermostability: 400 °C; Chemical stability: 1 d in 12 M HCl and 1 M NaOH.	[98]
C–N	β-ketoenamine 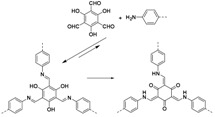	Crystallinity: Good; Thermostability: 350 °C; Chemical stability: More than 7 d in boing water, 9 M HCl and 9 M NaOH.	[99]
	β-ketoenamine 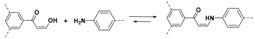	Crystallinity: Moderate; Thermostability: 300 °C; Chemical stability: 7 d in hot water (50 °C) and 9 M HCl; partial hydrolysis in 9 M NaOH.	[100]
	Azine 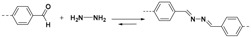	Crystallinity: Moderate; Thermostability: 250 °C; Chemical stability: 1 d in water, 1 M HCl, 1 M NaOH and organic solvents.	[101]
	Phenazine 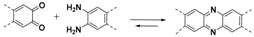	Crystallinity: Moderate; Thermostability: More than 1000 °C; Chemical stability: 1 d in water, 1 M HCl, 1 M NaOH and organic solvents.	[102]
	Squaraine 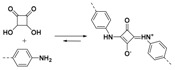	Crystallinity: Moderate; Thermostability: 300 °C; Chemical stability: 1 d in water, 1 M HCl, 1 M NaOH and organic solvents.	[103]
	Viologen 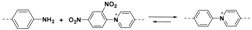	Crystallinity: Moderate; Thermostability: 400 °C; Chemical stability: 3 d in boiling water and 6 M HCl; sensitive to 1 M NaOH.	[104]
	Triazine 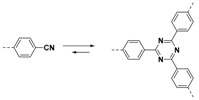	Crystallinity: Poor; Thermostability: 400 °C; Chemical stability: High stability (no precise description).	[105]
	Melamine 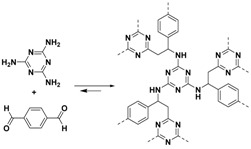	Crystallinity: Poor; Thermostability: 400 °C; Chemical stability: Stable in water and common organic solvents.	[106]
C–C	C–C 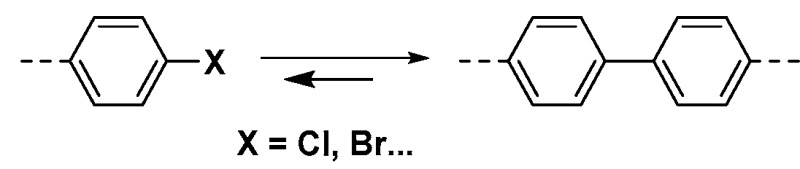	Crystallinity: No reported; Thermostability: 250 °C; Chemical stability: No reported.	[107]
	C=C 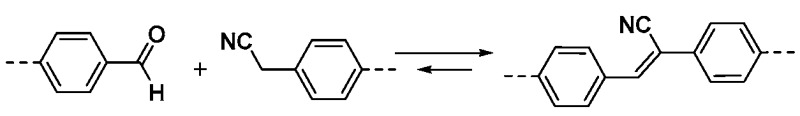	Crystallinity: Moderate; Thermostability: 350 °C; Chemical stability: High stability (no precise description).	[108]
B–N	Borazine 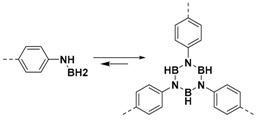	Crystallinity: Good; Thermostability: 420 °C; Chemical stability: No reported.	[109]
N–N	Azodioxide 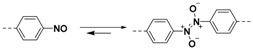	Crystallinity: Monocrystalline; Thermostability: Less than 130 °C; Chemical stability: No reported.	[110]

**Table 3:** Examples of COFs as drug carriers.

Year	COFs	Linkages	Pore Sizes (nm)	Morphologies	Model Drugs	Characters	Reference
2015	PI-COF-4 PI-COF-5	Imide	1.3 1.0	Rectangular; Length: Hundreds of nanometers	IBU, caffeine and captopril	DLE ^a^: 24 wt %; DRR: 95% for 6 days. DLE ^a^: 20 wt %; DRR: 95% for 6 days.	[63]
2015	COF-DhaTab	Imine	3.7	Submicron hollow spheres	DOX	DLE ^b^: 0.35 mg/g; DRR: 42% after 7 days at pH 5	[116]
2016	PI-2-COF PI-3-COF	Imine	1.4 1.1	Spherical nanoparticles; 50 nm	5-FU, IBU and captopril	DLE ^a^: 30 wt %; DRR: 85% for 5 days; Good biocompatibility.	[323]
2016	COF_ABBA_	Boroxine	2.1	Single-layer	CuPc	Photoresponsive release; No DLE nor DRR has been measured.	[266]
2016	TTI-COF	Imine	2.4	Elongated rods	Quercetin	DLE ^b^: fail to measure; DRR: fail to measure; Good biocompatibility.	[324]
2016	NCTP	Triazine	1.21	Spherical nanoparticles; 50–70 nm	DOX	DLE ^a^: 20 wt %; DRR: 60% for 2 days at pH 7.4 & 80% for 2 days at pH 4.8; Good biocompatibility.	[131]
2017	EDTFP-1	β-ketoenamine	1.5	Nanofiber; Diameter: 22–30 nm	TFP	TFP works as model drug as well as building block; Kill cancer cells by COFs themselves.	[328]
2017	TpASH-FA	β-ketoenamine	1.3	Nanosheets	5-FU	DLE ^b^: 12 wt %; DRR: 50% for 75 h at pH 7.4 & 75% for 75 h at pH 5; Good biocompatibility.	[64]
2017	PCTF PCTF-Mn	Triazine	0.8~2.7 0.7~4.2	Irregular nanoparticles; Plate-shaped	IBU	DLE ^a^: 19 wt %; DRR: 90% for 48 h. DLE ^a^: 23 wt %; DRR: 95% for 48 h.	[132]

^a^ measured by TGA; ^b^ measured by UV; DLE: Drug Loading Efficacy; DRR: Drug Release Rate; IBU: Ibuprofen; DOX: Doxorubicin; 5-FU: 5-Fluorouracil; CuPc: Copper Phthalocyanine; TFP: 2,4,6-Triformylphloroglucinol.
